# The evolution of skyrmions in Ir/Fe/Co/Pt multilayers and their topological Hall signature

**DOI:** 10.1038/s41467-018-08041-9

**Published:** 2019-03-06

**Authors:** M. Raju, A. Yagil, Anjan Soumyanarayanan, Anthony K. C. Tan, A. Almoalem, Fusheng Ma, O. M. Auslaender, C. Panagopoulos

**Affiliations:** 1 Division of Physics and Applied Physics, School of Physical and Mathematical Sciences, Nanyang Technological University, Singapore, 637371 Singapore; 2 Department of Physics, Technion, Haifa, 32000 Israel; 3 Data Storage Institute, Agency for Science, Technology and Research (A*STAR), 2 Fusionopolis Way, Singapore, 138634 Singapore; 4 Present Address: Institute of Materials Research and Engineering, Agency for Science, Technology and Research, Singapore, Singapore; 5 Present Address: Cavendish Laboratory, University of Cambridge, JJ Thomson Avenue, Cambridge CB3 0HE, Cambridge, United Kingdom

## Abstract

The topological Hall effect (THE) is the Hall response to an emergent magnetic field, a manifestation of the skyrmion Berry-phase. As the magnitude of THE in magnetic multilayers is an open question, it is imperative to develop comprehensive understanding of skyrmions and other chiral textures, and their electrical fingerprint. Here, using Hall-transport and magnetic-imaging in a technologically viable multilayer film, we show that topological-Hall resistivity scales with the isolated-skyrmion density over a wide range of temperature and magnetic-field, confirming the impact of the skyrmion Berry-phase on electronic transport. While we establish qualitative agreement between the topological-Hall resistivity and the topological-charge density, our quantitative analysis shows much larger topological-Hall resistivity than the prevailing theory predicts for the observed skyrmion density. Our results are fundamental for the skyrmion-THE in multilayers, where interfacial interactions, multiband transport and non-adiabatic effects play an important role, and for skyrmion applications relying on THE.

## Abstract (web summary)

The topological Hall Effect (THE) enhances our understanding of chiral spin textures such as skyrmions, but important aspects of the relationship are still unclear. Here the authors present a comprehensive picture for the spin texture evolution and corresponding THE signatures in a multilayer film using Hall transport and magnetic imaging.

## Introduction

Skyrmions are topologically protected, two-dimensional (2D), localized hedgehogs and whorls of spin^1^. Originally invented as a concept in field theory for nuclear interactions^2^, skyrmions are central to a wide range of phenomena in condensed matter^3–5^. Their realization at room temperature (RT) in magnetic multilayers^6–8^ has generated considerable interest, fueled by technological prospects and the access granted to fundamental questions. The interaction of skyrmions with charge carriers^1,8–12^ gives rise to exotic electrodynamics, such as the topological Hall effect (THE)^13,14^. The topological protection of skyrmions results from the quantization of their topological charge (*Q*_sk_), which counts the number of times the magnetization unit-vector **n**(**r**) covers the unit-sphere. *Q*_sk_ is determined by *B*_eff_(**r**), the *z*-component of the emergent magnetic field that corresponds to the Berry phase accumulated by a spin tracking **n**(**r**)^1,15,16^:

1 $$Q_{{\mathrm{sk}}} = \frac{1}{{4\pi }}{\int} d^2r{\mathbf{n}} \cdot \partial _x{\mathbf{n}} \times \partial _y{\mathbf{n}} \equiv \frac{1}{{{\mathrm{\Phi }}_0}}{\int} d^2rB_{{\mathrm{eff}}}({\mathbf{r}}) = 0, \pm 1, \pm 2, \ldots .$$

Here Φ_0_ = *h*/*e* is the flux quantum, *h* is Planck’s constant, −*e* is the electron charge. THE is the Hall response to *B*_eff_(**r**). When charge carriers flow through a conductor with their spins tracking the skyrmion spin texture, the topological-Hall resistivity (*ρ*_TH_) is^1^:

2 $$\rho _{{\mathrm{TH}}} = PR\prime_{\!\!0} n _{\mathrm{T}}{\mathrm{\Phi }}_0,$$

where *n*_T_ is the 2D density of total topological charge. Here $R_0{\!\!\prime}$ is an unknown Hall resistivity representing the effective density of charge contributing to THE. $R_0^\prime$ is usually taken to be *R*_0_^13,17–19^, the ordinary Hall coefficient, which is extracted from the high field slope of the Hall resistivity (*ρ*_*yx*_), and represents the total density of mobile charge. 0 < *P* < 1 is the spin-polarization of the charge carriers, and *B*_eff_(**r**) is manifested through Φ_0_. Assuming skyrmions are the sole carriers of topological charge |*Q*_sk_| = 1 implies *n*_T_ = *n*_sk_, the density of isolated skyrmions. Thus, within the adiabatic approximation, one expects a straightforward correlation between *ρ*_TH_ and *n*_sk_. From the first observations in B20 systems^13,14^ to the recent multilayers, THE has been used as an indicator for the presence of skyrmions^8,17^. However, a clear understanding of the effect is still lacking^20^, especially in technologically viable multilayer films^8,18,19^, where disorder and interface effects can play an important role^7,21,22^. In particular, and as we explain below, the THE features in these multilayers are subtle and call for careful analysis of the transport measurements, as we demonstrate in Supplementary Note 3 and Supplementary Note 4. In contrast, the situation in some B20 systems is straightforward as they show a distinguishable characteristic hump in *ρ*_*yx*_ as a function of applied magnetic field (*H*) and temperature (*T*), corresponding to a skyrmion lattice^23,24^. However, even in B20 systems such a feature does not appear always^25^. One of our main goals here is to elucidate the significance of the subtle features that characterize our multilayer and to test their relationship to skyrmions.

Using magnetic force microscopy (MFM) and transport measurements, we present a comprehensive picture of the evolution of magnetic textures and their THE signature in a multilayer film capable of hosting skyrmions from RT down to at least 5 K. We demonstrate the relationship between *n*_sk_ and |*ρ*_TH_| over a ≈200 K temperature range. As the applied field *H* is swept from saturation towards zero, we find that skyrmions aggregate in worm-like magnetic textures, which may carry large topological charge (*Q*_W_), and manifest as peaks in *ρ*_TH_. Quantitative modeling of these worm-textures uncovers qualitative agreement between *ρ*_TH_(*H*, *T*) and *n*_T_(*H*, *T*). Despite this, we find a large quantitative discrepancy indicating that the effect in multilayers is more involved.

## Results

### Multilayer system

Here we use sputtered [Ir(1)/Fe(0.5)/Co(0.5)/Pt(1)]_20_ (in parenthesis—thickness in nanometers; subscript denotes the number of repeats) multilayer films, with the composition chosen for exhibiting skyrmions across a large range of *T*. The RT characterization of the films indicates Dzyaloshinskii–Moriya interaction (DMI) *D* ≈ 2.0 mJ/m^2^, and exchange interaction *A* ≈ 11 pJ/m^8^. The effective magnetic anisotropy (*K*_eff_) varies in the range ≈0.2–0.01 MJ/m^3^ as we change *T* from 5 K to 300 K (Supplementary Note 2). As demonstrated here, control over *n*_sk_ through variation of *T* is the key for unambiguous identification of the skyrmion THE signature. Without this control, the correspondence between *n*_sk_ and the subtle THE signal, which we establish by direct imaging, is impossible to uncover. In contrast to the B20 compounds, which host lattices of tubular Bloch-skyrmions^26^, multilayers sustain skyrmions with tunable properties, and offer smoother integration with existing spintronic technologies. Spin textures in multilayers are influenced by interlayer dipolar and exchange interactions, magnetic frustration^27^, and granularity^7^, which can pin, stabilize, and deform the spin textures, and result in coupled pancake-skyrmions with different topologies^22,27^. This complexity, and associated tunability, provide means for exploring the interplay between disorder, interactions, and topology.

### Qualitative agreement between residual Hall signal and the magnetic textures

The magnetoresistance and Hall effect were measured using a lock-in with non-perturbative current densities (≈10^5^ A/m^2^). The presence of skyrmions is associated with an additional component in the measured *ρ*_*yx*_^13,14,17^. This contribution can be quantified by resolving *ρ*_*yx*_ into the ordinary (*R*_0_*H*) and anomalous [*R*_S_*M*(*H*)] Hall components^13,17–19^, and *ρ*_TH_:

3 $$\rho _{yx}(H) = R_0H + R_{\mathrm{S}}M(H) + \rho _{{\mathrm{TH}}}(H).$$

**Fig. 1:**
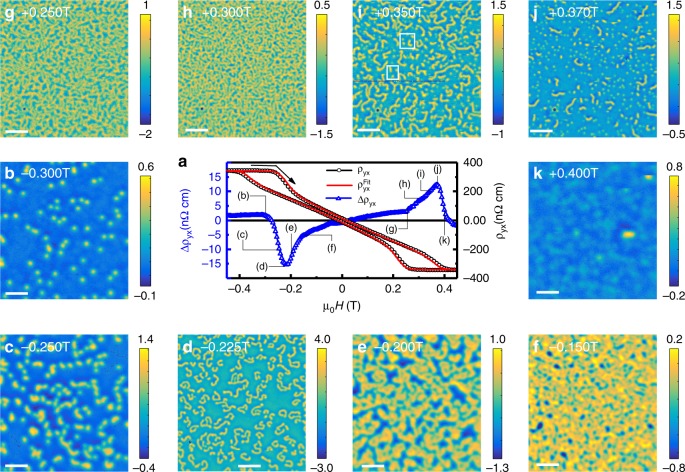
Evolution of magnetic textures and THE with *H* at *T* = 5 K. **a** *ρ*_*yx*_(*H*), $\rho _{yx}^{fit}(H)$, and the residual ${\mathrm{\Delta }}\rho _{yx}(H) = \rho _{yx}(H) - \rho _{yx}^{fit}(H) \approx \rho _{{\mathrm{TH}}}(H)$. The black arrow indicates the field sweep direction for Δ*ρ*_*yx*_ and MFM, while *ρ*_*yx*_ and $\rho _{yx}^{fit}$ are shown for both sweep directions. **b**–**k** Selected MFM scans [full sequence in Supplementary Figure 16, scan-height *h* = 75, 60, 40, 60, 65, 50, 60, 50, 45, 60 nm for each of **b**–**k**; color bars give the range of Δ*f*, scale bars are 1 μm]. Frames in (i) mark features in focus in Fig. 3b–g

We estimate *ρ*_TH_(*H*) by Δ*ρ*_*yx*_(*H*), the residual of the fit of *ρ*_*yx*_(*H*) to $\rho _{yx}^{fit}(H) = R_0H + R_{\mathrm{S}}M(H)$, which also yields *R*_0_ and *R*_S_ (Supplementary Figure 7). The accuracy of Δ*ρ*_*yx*_ is ensured by calibrating field offsets to avoid artifacts resulting from using different measurement setups (Supplementary Note 3 and Supplementary Figure 2). Our conservative estimate for the overall error in Δ*ρ*_*yx*_, including a contribution from data analysis, is ±2 nΩ ⋅ cm, corresponding to the non-zero residual signal beyond saturation, where there are no skyrmions. Figure 1a shows Δ*ρ*_*yx*_(*H*) at 5 K, with the overall features persisting to at least 300 K^8^.

The features of Δ*ρ*_*yx*_(*H*) in Fig. 1a are subtle. Here, we use MFM to determine the correspondence of these features with the magnetic texture. For this we followed the same field sweep from saturation as we did for Δ*ρ*_*yx*_(*H*) and magnetization [*M*(*H*)]—the images were acquired at field increments as *H* was swept (Supplementary Note 1). Figure 1b–k shows the result for 5 K. Overall, we observe a similar evolution of the magnetic textures at *T* = 50, 100, 150, 200 K (Supplementary Figures 17 to 20).

We begin by comparing the magnetic textures to Δ*ρ*_*yx*_(*H*). Beyond saturation, MFM shows the null signal expected for a polarized ferromagnet [Supplementary Figure 16(a)]. This is accompanied by the suppression of Δ*ρ*_*yx*_ (Fig. 1a) as expected for the topologically trivial polarized state. The onset of Δ*ρ*_*yx*_ commences at *μ*_0_*H* ≈ −0.3 T with the nucleation of sub-100 nm magnetic domains (Fig. 1b), which we identify as Néel-skyrmions^21^. By *μ*_0_*H* ≈ −0.25 T (Fig. 1c) the increasing *n*_sk_ corresponds to a substantial Δ*ρ*_*yx*_ as expected from |*ρ*_TH_| ∝ *n*_sk_^1^. Also, as *n*_sk_ increases, skyrmions aggregate to form worm-like features (Fig. 1c). By *μ*_0_*H* ≈ −0.225 T, images show only worms (Fig. 1d, e). Surprisingly, at this field Δ*ρ*_*yx*_ peaks, suggesting a significant contribution from the worms which may have nontrivial topology. Meanwhile, the dense textures at intermediate *H* (Fig. 1f–h, and Supplementary Figures 17 and 18) correspond to reduced, yet finite, Δ*ρ*_*yx*_. Careful inspection of such scans reveals worm-like features, to which we attribute the finite magnitude of Δ*ρ*_*yx*_ (Supplementary Note 7).

As *H* = 0 is approached, the worms evolve into labyrinthine helical stripes, and proliferate at the expense of the polarized background. This is coincident with the suppression of Δ*ρ*_*yx*_, highlighting the close relationship between Δ*ρ*_*yx*_ and the magnetic texture. As *H* is increased towards positive saturation, the labyrinthine stripes evolve into worms, skyrmions, and eventually a uniformly polarized phase (Fig. 1i–k). For a texture with an opposite topological charge, the sign of Δ*ρ*_*yx*_ is reversed when *H* > 0. However, the MFM contrast does not change, due to the reversal of the tip magnetization near 0.1 T.

The distinct field ranges for isolated skyrmions (near saturation), worms (negative peak in Δ*ρ*_*yx*_), and their coexistence (positive peak in Δ*ρ*_*yx*_) (cf. Supplementary Note 6), offer a unique opportunity to compare the magnetic texture with Δ*ρ*_*yx*_. In particular: (i) Does Δ*ρ*_*yx*_ track *n*_sk_? (ii) How do worms produce such a large Δ*ρ*_*yx*_ ? (iii) Is there quantitative consistency between Δ*ρ*_*yx*_ and *n*_T_?

**Fig. 2:**
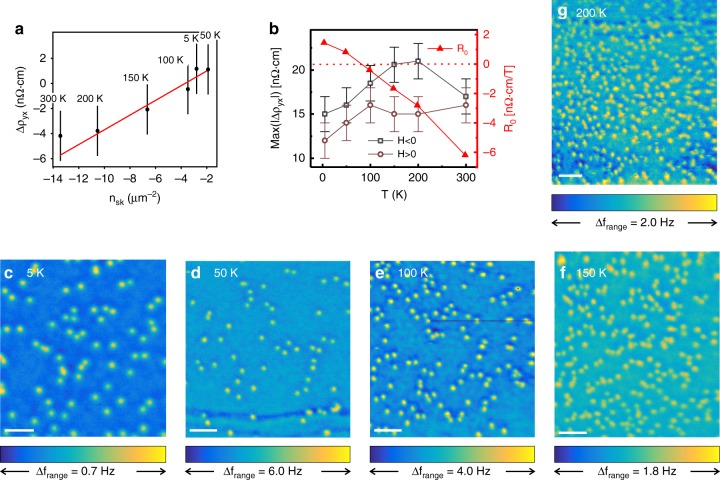
Temperature dependence of *n*_sk_, Δ*ρ*_*yx*_, and *R*_0_. The sign of *n*_sk_ is chosen to match Δ*ρ*_*yx*_. **a** Δ*ρ*_*yx*_ as a function of *n*_sk_ for different temperatures at *μ*_0_*H* = −0.3*T*. Labels show the measurement temperature. Error-bars for Δ*ρ*_*yx*_ represent a conservative estimate of the systematic error. Errors for *n*_sk_ are smaller than the symbols. The line is a linear fit (slope 0.6 ± 0.1 nΩ · cm/μm^−2^, intercept 2.2 ± 0.8 nΩ · cm). **b** Left axis: magnitude of the THE peaks (*H* < 0—squares, *H* > 0—circles). Right axis: the fit parameter *R*_0_(*T*) from Eq. (3) (triangles). **c**–**g** MFM scans showing isolated skyrmions for *n*_sk_(*T*) in **a**. [For **c**–**g** scan-height *h* = 75, 40, 40, 100, 40 nm, (**c**) is Fig. 1b, scale bars are 1 μm]

To address (i) we exploit *n*_sk_(*T*), which increases by an order-of-magnitude when we increase *T* from 5 to 200 K (Fig. 2). We attribute this proliferation to the suppression of *K*_eff_ with temperature^8^. Interestingly, skyrmion size is only weakly *T*-dependent and does not change significantly within our resolution, not unlike theoretical predictions^28^. However, further experiments with similar imaging conditions are required to confirm the size variation. Importantly, we find that Δ*ρ*_*yx*_(*T*) tracks *n*_sk_(*T*) over the entire range (Fig. 2a), and gives ≈0.6 nΩ ⋅ cm per skyrmion/μm^2^. This correlation between skyrmion nucleation and the emergence of Δ*ρ*_*yx*_ strongly points towards the topological origin of the residual signal.

### Magnetic worms and their topological charge

**Fig. 3:**
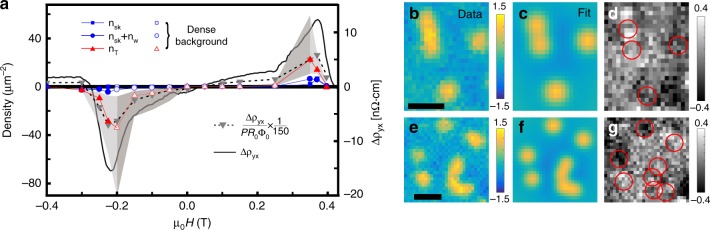
Comparison between Δ*ρ*_*yx*_ and the signed density of topological charge at 5 K. **a** Right axis: Δ*ρ*_*yx*_ (solid line). Left axis: *n*_sk_ (squares), *n*_sk_ + *n*_w_ (circles), *n*_T_ (triangles), and Δ*ρ*_*yx*_/(*PR*_0_Φ_0_) (cf. Eq. (2), inverted triangles with dotted line) using *P* = 0.56^43^ and *R*_0_ from Fig. 2b. For *n*_T_, each worm is assigned several skyrmions |*Q*_W_| by fit, and each isolated skyrmion is counted once. Empty symbols indicate points with a lower confidence, that result from counting worms in a dense background (Supplementary Note 7). Shaded area shows confidence bounds for *n*_T_ resulting from fit details (Supplementary Note 7). **b**, **e** Zooms on areas in Fig. 1i with skyrmions and isolated worms. **c**, **f** Results of MEFS fits wherein all skyrmions, including those assigned to worms, are treated as identical. The fit peak height ≈1.15 Hz, full width at half maximum (FWHM) ≈100 nm, and |*Q*_W_| = 2 in (**c**) and |*Q*_W_| = 4 in **f**. **d**, **g** Difference plots between **b** and **c**, and between **e** and **f**, showing the quality of the fit. Circles give the locations of the skyrmions in the fit. [The scale bars in **b** and **e** are 200 nm]

Having established the direct correspondence between *n*_sk_ and Δ*ρ*_*yx*_, we now examine the worms. Though the presence of worms is expected in systems with competing interactions, such as in ferromagnetic films^29^, their topological role is not obvious. In early MFM work on Bloch skyrmions in a B20 compound^26^, helical stripe domains resulting from merging skyrmions were described by two half-skyrmions connected by a topologically trivial straight domain^26^, and hence a topological charge of *Q*_w_ = ±1. This motivates us to examine whether worms carrying a topological charge given by *Q*_sk_ = ±1 can describe our results. We therefore plot *n*_sk_ + *n*_w_ (*n*_w_ is the number of worms per unit area) in Fig. 3a, with the sign chosen from the sign of Δ*ρ*_*yx*_. As the plot shows, both *n*_sk_(*H*) and *n*_sk_(*H*) + *n*_w_(*H*) do not track Δ*ρ*_*yx*_(*H*). This calls for a closer look at the topological nature of the worms.

The following analysis of the topological charge of worms is motivated by sequences like Fig. 1b–d [also in Supplementary Figures 17(n)–(o)], which suggest that worms result from skyrmions clustering as *n*_sk_ increases. The transition of worms into typical stripe domains with |*Q*_w_| = 1 requires a complete unwinding of their internal spin structure. The energy barrier for this suggests that the effective topological charge should be at least equal to the total number of skyrmions that form a worm (i.e., |*Q*_w_| > 1). While skyrmions are expected to repel each other on very short length-scales because of exchange coupling^30^, clusters can be stabilized by attraction on an intermediate scale, due to exchange frustration^31^.

Here our recent work, a magnetic multipole expansion of the field from skyrmions (MEFS)^21^, provides a direct method to associate an effective *Q*_w_ with each worm. Figure 3b–g shows two typical examples where we fit the measured signal from worms by trains of skyrmions. Such images, which contain both skyrmions and worms, provide the foundation for this kind of analysis—the isolated skyrmions serve to constrain the fit amplitude per skyrmion, and improve the accuracy (Supplementary Note 7). Meanwhile, the analysis of images containing only worms (Fig. 1d) hinges on skyrmions–skyrmion repulsion on a length scale comparable to their radius^30^. Therefore, the number of skyrmions clustered in a worm is determined by the total length of the worm, and the typical radius of skyrmions (≈40 nm^21^). For images with densely packed features (e.g., Fig. 1f–h) identifying and extracting the worms themselves requires additional image processing, for which we employ a deep-learning-model-based algorithm that extracts features relevant for classification from supplied examples (Supplementary Note 7). We note however, that while this analysis provides additional evidence for the correlation between magnetic textures and the Δ*ρ*_*yx*_ that we extract, our main conclusion does not rely on it. In all cases, our analysis allows us to compute the total *n*_T_(*H*) from ∑*Q*_w_ + ∑*Q*_sk_, where ∑*Q*_w_ is the topological charge of a worm after assigning an appropriate number of skyrmions to it, and we assume that all skyrmions (including those in worms) have *Q*_sk_ = ±1, with the sign chosen to match the sign of Δ*ρ*_*yx*_.

To further verify our assertion of worms as trains of skyrmions, we have performed basic simulations of magnetization and the resulting evolution of magnetic textures in our multilayer stack. Simulations were performed using the MuMax^3^ package^32^ at *T* = 0 K for two different sets of magnetic parameters relevant to the magnetization of the stacks at different temperatures. In the simulations the field is swept incrementally towards saturation from *H* = 0. The magnetic texture in the simulations starts from labyrinthine stripes and evolves into worms, skyrmions and eventually a fully polarized state, in agreement with our experimental results. A complete set of simulated MFM images is shown in Supplementary Figures 10 and 11.

**Fig. 4:**
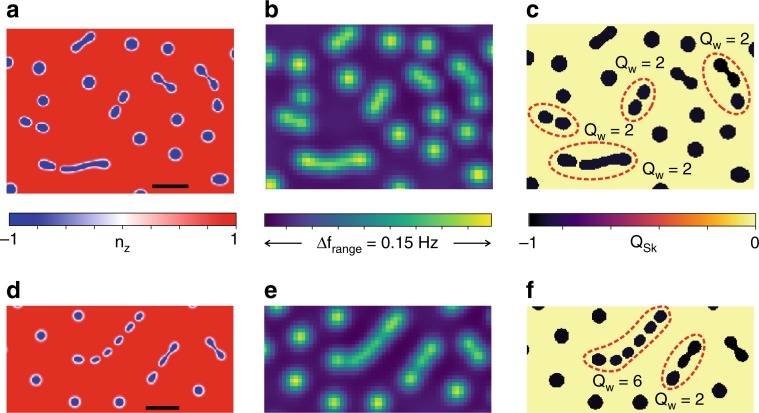
Micromagnetic simulations of magnetic textures (Supplementary Note 5) for two sets of magnetic parameters corresponding to 5 and 200 K: **a**–**c** *A* = 11 pJ/m, *D* = 2 mJ/m^2^, *M*_*s*_ = 1.34 MA/m, *K*_eff_ = 0.2 MJ/m^3^, and **d**–**f** *A* = 11 pJ/m, *D* = 2 mJ/m^2^, *M*_*s*_ = 1.16 MA/m, *K*_eff_ = 0.05 MJ/m^3^. **a**, **d** 2D maps of the out-of-plane magnetization (*n*_*z*_) at 0.230 T. **b**, **e** Simulated MFM images at *h* = 40 nm obtained by integrating the stray field from the micromagnetic simulations and convolving with a model for the tip (Supplementary Note 5). **c**, **f** Contribution to the topological charge from each domain, calculated using Eq. (1) (Supplementary Note 5). Dashed lines highlight trains of skyrmions which appear as individual worms in the MFM scans. [The scale bars in **a** and **d** are 200 nm]

Figure 4 shows representative simulated maps of *n*_*z*_(**r**), the corresponding MFM images, and the contribution to *Q*_sk_ from each individual domain. Dashed domains in Fig. 4c, f mark skyrmion trains which appear as worms in the MFM scans (Fig. 4b, e), clearly indicating |*Q*_W_| > 1. Here, we point out that while these results are in qualitative agreement with the experimental evolution of magnetic textures and the formation of worms, the simulations do not account for disorder and thermal effects in the multilayers. Addition of such effects and a full theoretical treatment is necessary for a deeper understanding of skyrmion trains and skyrmion–skyrmion interactions.

The qualitative match between *n*_T_(*H*) and Δ*ρ*_*yx*_(*H*) (Fig. 3a), and the formation of worms from the clustering of skyrmions, as suggested by our micromagnetic simulations, reinforces our modeling of worms as trains of skyrmions. These results provide additional evidence that Δ*ρ*_*yx*_ results from skyrmion textures and is thus topological in nature, indicating Δ*ρ*_*yx*_ ≈ *ρ*_TH_. Our observations on the emergence of worms with a potentially high topological number, previously not noticed experimentally, indicate they are distinct from trivial spin spirals, and form an essential part of the phase diagram for multilayer skyrmions^33^. The presence of worms in a tunable multilayer offers a platform for studying skyrmion–skyrmion interactions over a wide parameter range, as well as applications, such as skyrmion racetracks^7^.

### Quantitative agreement between transport and imaging

Having explored the nontrivial topology of the worms and a qualitative match between *ρ*_TH_(*H*, *T*) and *n*_T_(*H*, *T*), we examine the quantitative match. As we show in Fig. 3a, the density of topological charge estimated from THE [Δ*ρ*_*yx*_/(*PR*_0_Φ_0_), Eq. (2)] indicates a two-orders-of-magnitude discrepancy. This implies that simply using Eq. (2) with $R_0^\prime = R_0$ to understand the topological signatures of chiral magnetic textures in multilayer skyrmion-hosts does not yield a comprehensive description of the measured Δ*ρ*_*yx*_.

A possible culprit is the assignment $R_0^\prime = R_0$ in Eq. (2)^34^, which is justified for a single band material. This is not the case here: Bulk Fe and Co, the ferromagnetic ingredients of our multilayer stack, have several active electron and hole bands^35^. In such materials *R*_0_ is suppressed because electrons and holes, which experience the same *H*, compensate each other’s contributions. The cancellation estimated from values reported for bulk Fe^35^ indicate suppression of *R*_0_ by an order of magnitude from the separate contributions of individual bands, partially addressing the discrepancy (Supplementary Note 8). Importantly, the cancellation may not happen in the same way for *B*_eff_—the Berry-phase can act differently on charge carriers from different bands^23^, with an associated sensitivity to occupation^36^. The fact that the peak value of Δ*ρ*_*yx*_(*H*) changes by only ≈25% with *T* despite the sign change of *R*_0_(*T*) (Fig. 2b), probably because of small variations of the occupations of the compensating bands, further confirms that $R_0^\prime \ne R_0$. In this case, using *R*_0_ in Eq. (2) underestimates *ρ*_TH_^18,19^, although once $R_0^\prime$ is determined this fundamental equation may still be used. For this it is essential to account for the electronic band structure of the chiral magnet.

Finally, we comment on the nature of spin transport in multilayers. Here, transport is influenced by various length scales such as the thickness of individual layers, their mean free path (*l*), charge and spin conductivities, spin diffusion length (*l*_*s*_), and the size of the magnetic textures—including the domain walls and their chirality. In our case, the radius of the skyrmions is ≈40 nm and the domain wall width ≈7–14 nm $\left( {\sqrt {A/K_{{\mathrm{eff}}}} } \right)$, where, *A* ≈ 11 pJ/m and ≈0.2–0.05 MJ/m^3^. These length scales are comparable to the transport length scales reported for single thin layers (*l*_Co_ ≈ 5 nm and *l*_Pt_ ≈ 13 nm, *l*_*s*−*Pt*_ ≈1−10 nm)^37^, indicating their relevance to the relationship between *ρ*_TH_ and *n*_sk_. Spin scattering from interfaces and magnetic textures such as domain walls may cause spin-flip scattering, resulting in a non-adiabatic, spin independent transport^38–40^. As a result of the disordered skyrmion configurations, the charge carriers experience an inhomogeneous emergent field, in contrast to charge carriers in bulk systems which experience a systematically varying uniform emergent field due to the ordered arrangement of skyrmion textures. Notably, spin scattering in multilayer systems and its contribution to the anomalous Hall effect is dominated by skew scattering^41^. Hence, a better understanding of this contribution may help resolve the discrepancy between the observed and the expected THE signal^38–40,42^.

## Discussion

Our complementary imaging and electrical transport studies provide clear evidence for the correlation between Δ*ρ*_*yx*_ and the topology of the magnetic texture in technologically viable magnetic multilayers. Furthermore, we elucidate the complexity of the Berry-phase associated with the electrical fingerprint of chiral magnetic textures in those skyrmions hosting platforms. For a comprehensive understanding and in order to utilize THE emerging from magnetic skyrmions, it is imperative to consider (a) the band structure contributing to THE, (b) the possibility of |*Q*_sk_| > 1^27^ and skyrmion–skyrmion interactions^31^, (c) the coupling of skyrmions across layers and complex magnetic textures in buried interfaces^22^, (d) the contribution from topologically trivial chiral configurations driven by magnetic spin-frustration^20^, and (e) the validity of the commonly assumed adiabatic approximation^20^.

## Supplementary information

Supplementary information file

## Data Availability

The authors declare that the data supporting the findings of this study are available within the paper, and its Supplementary Information.
