# Supplementary material for: The evolution of skyrmions in Ir/Fe/Co/Pt multilayers and their topological Hall signature
Source: Nat Commun. 2019 Mar 6;10:696. doi: 10.1038/s41467-018-08041-9 (PMC6403237; doi:10.1038/s41467-018-08041-9)
Supplement: Supplementary file 1 — Supplementary information file [file 41467_2018_8041_MOESM1_ESM.pdf]

Supplementary Information for

The evolution of skyrmions in Ir/Fe/Co/Pt multilayers and their topological Hall signature

M. Raju,[1*] A. Yagil,[2*] Anjan Soumyanarayanan,[3,1] Anthony K. C. Tan,[1,3]

A. Almoalem,[2], Fusheng Ma[1], O. M. Auslaender,[2†] C. Panagopoulos[1†]

[1]Division of Physics and Applied Physics, School of Physical and Mathematical Sciences, Nanyang Technological University, 637371 Singapore

[2]Department of Physics, Technion, Haifa 32000, Israel

[3]Data Storage Institute, Agency for Science, Technology and Research (A*STAR), 2 Fusionopolis Way, 138634 Singapore

[*]These authors are equal contributors.

[†]To whom correspondence should be addressed; E-mail: ophir@physics.technion.ac.il, christos@ntu.edu.sg

CONTENTS

List of Supplementary Figures 2

Supplementary Note 1 Experimental Methods 3

Supplementary Note 2 Magnetic Properties 4

Supplementary Note 3 Magnetic Field Calibration 4

Supplementary Note 4 Hall Transport 5

A Topological Hall Analysis 5

B Scaling of ρ_{yx} with ρ_{xx} and validity of the topological Hall effect analysis 7

C Temperature Dependence of the Topological Hall Effect 10

D Evolution of the Topological Hall and Magnetic Textures at 50 K 12

E Metastability and Manipulation of Magnetic Textures 12

Supplementary Note 5 Micromagnetic Simulations 14

A Simulated MFM Images 15

B Topological Charge of Simulated Worms 15

Supplementary Note 6 MFM Results 16

A MFM Scans Near the Peaks of $\Delta\rho_{yx}$ 17

Supplementary Note 7 Image Analysis 18

A Labeling . . . 19
B Fitting . . . 20
C Worms in a Background of Dense Domains . . . 21
Supplementary Note 8 Estimate of the Ordinary Hall Coefficient . . . 26
Supplementary References . . . 26
Appendix – MFM Images as a Function of Field and Temperature . . . 28

LIST OF SUPPLEMENTARY FIGURES

1 The magnetization and its temperature dependence . . . 5
2 Magnetic field calibration . . . 6
3 Extraction of the topological Hall signal . . . 7
4 The role of ρ_{xx} and the extracted residual signal . . . 8
5 Scaling dependency of ρ_{yx} on ρ_{xx} as a function of T at fixed H . . . 9
6 Scaling dependency of ρ_{yx} on ρ_{xx} as a function of H at a fixed T . . . 10
7 Temperature dependence of the magnetization and the Hall effect . . . 11
8 Comparison between $\Delta\rho_{yx}$ and the signed density of topological charge at 50 K 13
9 Examples of metastability and the influence of the MFM tip on magnetic texture . . . 14
10 Results of micromagnetic simulations . . . 16
11 Results of micromagnetic simulations . . . 17
12 MFM scans on $H < 0$ THE peak vs T . . . 18
13 MFM scans on $H > 0$ THE peak vs T . . . 19
14 Application of the deep learning model on a test set image . . . 23
15 Application of the deep learning model on experimental data . . . 25
16 MFM scans at $T = 5$ K . . . 28
17 MFM scans at $T = 50$ K . . . 29
18 MFM scans at $T = 100$ K . . . 30
19 MFM scans at $T = 150$ K . . . 31
20 MFM scans at $T = 200$ K . . . 31

Supplementary Note 1. EXPERIMENTAL METHODS

Multilayer films consisting of Ta(3)/Pt(10)/[Ir(1)/Fe(0.5)/Co(0.5)/Pt(1)]$_{20}$/ Pt(2) (layer thickness in nm, in parentheses) were deposited on thermally oxidized 100 mm Si wafers by DC magnetron sputtering at RT, using a Chiron^{TM} UHV system manufactured by Bestec GmbH. The base pressure prior to film deposition was 1×10^{-8} Torr, and a working pressure of 1.5×10^{-3} Torr was maintained during the deposition by Ar sputtering gas. Buffer layers of Ta(3)/Pt(10) were deposited before the active stack for better adhesion and film texture. Additional capping by Pt(2) was added to protect the stacks from unwanted oxidation. Film texture, surface roughness and interface quality were verified by X-ray diffraction (XRD), atomic force microscopy (AFM), and X-ray reflectivity (XRR) [1]. Magnetization [$M(H)$], Hall effect, and magnetic force microscopy (MFM) characterization were performed on different pieces of the same wafer.

The magnetization [$M(H)$] measurements were carried out using a superconducting quantum interference device (SQUID) with a magnetic property measurement system (Quantum Design MPMS XL). Magnetotransport measurements were carried with a lock-in technique using small current densities (as low as 10^5 A/m^2) to avoid current induced changes in the magnetic textures. The data was acquired at various temperatures (T) between 5 K to 300 K through a full hysteresis cycle, with 5 mT steps between the negative saturation field ($H_{\rm S}^-$) and the positive saturation field ($H_{\rm S}^+$) after saturation at large fields (+4 T). Measurements were carried out using an physical property measurement system (Quantum design, PPMS 6000), complemented by custom built variable temperature insert (VTI) housed in a high field magnet [1].

The magnetic imaging experiments were conducted using a cryogenic frequency modulated MFM system [2]. The sample was first stabilized at a given temperature ($5 - 200$ K) and then magnetized in the out of plane direction by applying $\mu_0 H = -0.5$ T, well beyond the saturation field (H_s^{OP}). After saturation, MFM images were acquired at field increments as H was swept from $H_{\rm S}^-$ to $H_{\rm S}^+$. Images were acquired by scanning the magnetic MFM tip parallel to the film surface at a constant scan height h without feedback, while recording changes of the resonant frequency (Δf) of the cantilever from its natural value (f_0) due to magnetic forces acting on the tip [3]. These shifts provide information on magnetic texture through the magnetic forces acting on the tip. In the limit of small oscillation amplitude and

small frequency shift [2]: $\Delta f \approx f_1 - (f_0/2k_0)(\partial F_z/\partial h)$, where k_0 is the spring constant of the cantilever [4], and f_1 is a constant offset. It is possible to account for the finite oscillation amplitude [5], but as we have demonstrated in recent work [6], this is not needed to describe the signal phenomenologically. It is important to note that the signal magnitude strongly depends on the tip-sample separation height h. Therefore, comparing the variation in Δf between scans is only meaningful if they were obtained at very similar values of h.

Supplementary Note 2. MAGNETIC PROPERTIES

Supplementary Fig. 1(a) shows the SQUID $M(H)$ magnetization loops recorded in out-of-plane (OP) and in-plane (IP) applied field at 100 K, while Supp. Fig. 1(b) shows the variation of the saturation magnetization and effective out-of-plane anisotropy (K_{eff}) with temperature. K_{eff} is determined from the difference in the saturation fields of the OP and the IP magnetization loops: $K_{\mathrm{eff}} = \mu_0 M_{\mathrm{S}} \left(H_s^{IP} - H_s^{OP}\right)/2$. H_s^{OP} was taken as the average saturation field of sweep-up and sweep-down branches [7]. The details of magnetic parameters such as D, A, K_{eff} and skyrmion properties for various thicknesses of Fe/Co have been reported previously [1].

Supplementary Note 3. MAGNETIC FIELD CALIBRATION

The estimated topological Hall effect (THE) in these multilayers is $5\% - 10\%$ of the total Hall signal, and any field offsets between the experimental setups can often appear as residual signals. In recent work, we have established the robustness of the residual signal at RT, and the results were found to be consistent across different measurement setups [1]. In this work using MPMS and PPMS, to avoid spurious residual signals, repeated measurements were carried out to verify the magnetic field offset using a Pd reference sample (Quantum Design, Serial no.: Pd#090721 − 04, weight: 0.2812 g, susceptibility $\chi_g = 5.24 \times 10^{-6}$ cm^3/g) and a Hall sensor (Lakeshore, model: XHGT-9060, serial no.: 53710, sensor type: InAs) respectively. Supplementary Figs. 2(a) and (c) show the set field vs measured field and their offset for MPMS and PPMS respectively. The field offset data presented in Supp. Figs. 2(b) and 2(d) represent the repeated field calibration runs carried out for different field sweep protocols. The variation in the field offsets is below ± 1 mT in the field range (± 0.6 T)

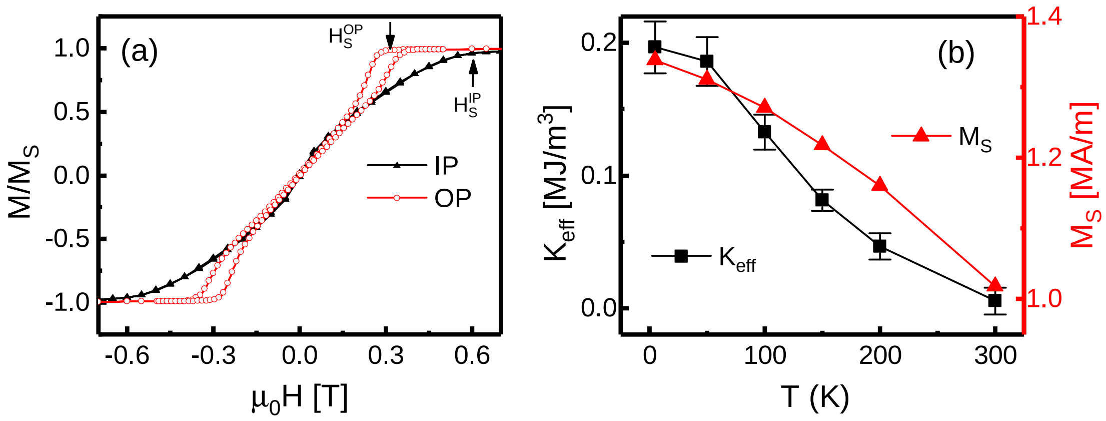

SUPP. FIG. 1. The magnetization properties of [Ir(1)/Fe(0.5)/Co(0.5)/Pt(1)]$_{20}$. **(a)** Magnetization loops recorded at 100 K in out-of-plane (OP) and in-plane (IP) applied fields. The effective out-of-plane anisotropy (K_{eff}) is determined from the difference in saturation fields ($H_s^{IP} - H_s^{OP}$). **(b)** Temperature dependence of the effective out-of-plane anisotropy and the saturation magnetization. Error bars in K_{eff} represents the systematic error in determining the magnetic saturation field and the magnetization.

relevant to the samples studied here. To avoid any field offset discrepancies resulting from the variation in the field protocols, the calibration runs were performed using the same protocols used for measuring the actual samples.

Supplementary Note 4. HALL TRANSPORT

A. Topological Hall Analysis

In case of B20 skyrmion lattices, such as in bulk crystals of MnSi and FeGe, the measured raw Hall resistivity (ρ_{yx}) shows a characteristic hump [8, 9] which correlates with the emergence of a skyrmion lattice. This characteristic feature in B20 clearly differentiates the transport data from magnetization. However, as mentioned in the main text, this feature does not always appear. In our multilayer case the characteristic features in the raw Hall data are subtle and need more careful inspection. As described in the main text, the topological Hall signal is extracted as the residual of the fit of ρ_{yx} to $\rho_{yx}^{fit} = R_0 H + R_{\text{S}} M(H)$. It is thus important to establish the validity of this residual signal and the analysis employed. Here, by comparing the first derivatives of the measured data $-d\rho_{yx}^{\text{norm}}/dH$ and dM^{norm}/dH, we show

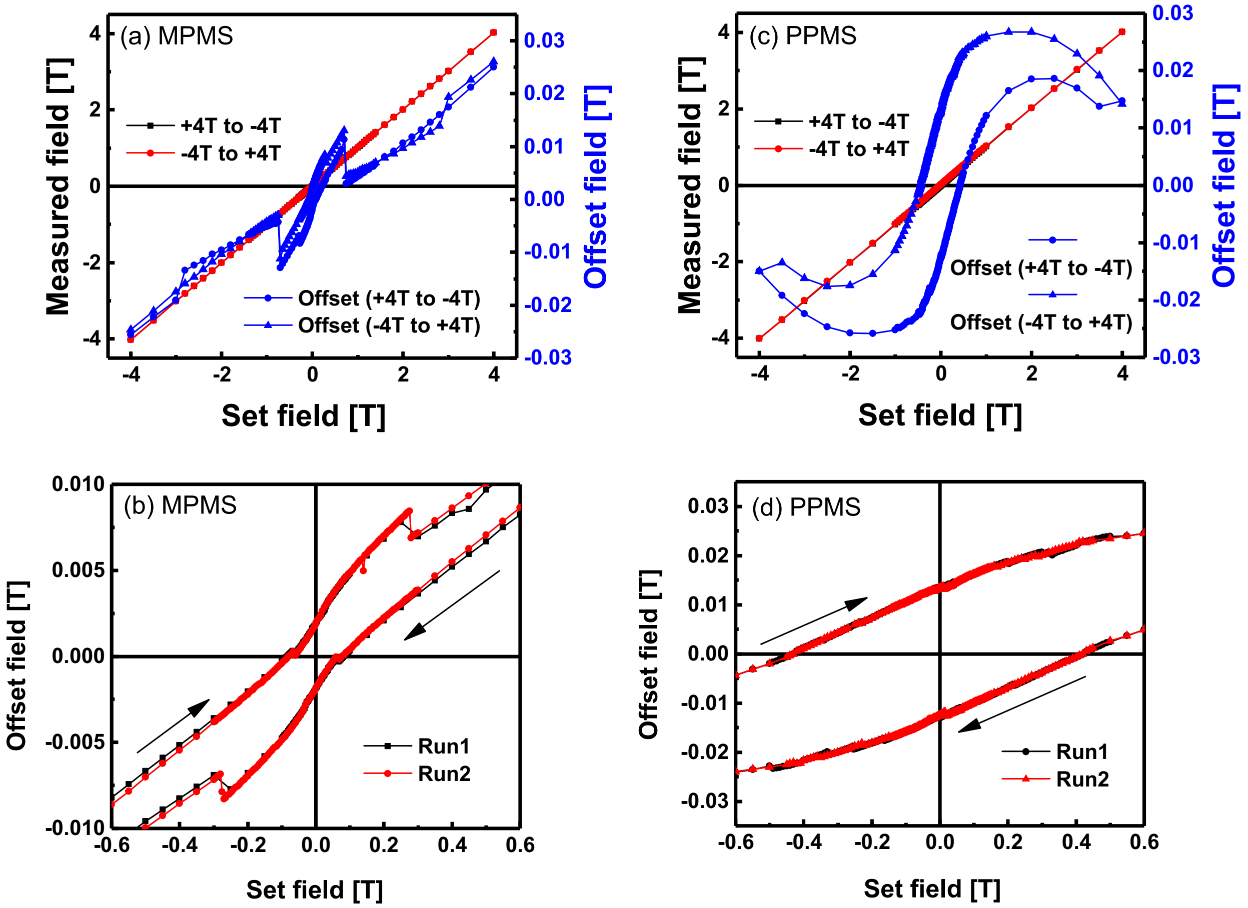

SUPP. FIG. 2. Magnetic field calibration for PPMS and MPMS. **(a)** Set vs measured magnetic field and their offset [defined as (set field)–(measured field)] using a Pd reference sample in MPMS. **(b)** Magnetic field offset in MPMS for two different field protocols. **(c)** Set vs measured magnetic field and their offset [defined as (set field)–(measured field)] using a Hall sensor in PPMS and **(d)** the magnetic field offset in PPMS for two different field protocols. [The arrows in panels (b) and (d) indicate the field sweep direction.]

that the observed residual signal indeed results from the additional component captured in the transport data [1]. Supplementary Figs. 3(a),(b) show the as-measured and magnetic-field offset-corrected magnetization and transport data for $[Ir(1)/Fe(0.5)/Co(0.5)/Pt(1)]_{20}$ recorded at 5 K. As shown in Supp. Fig. 3(c), the first derivatives clearly show the distinct peaks separated by 20 mT (blue and red dashed lines) indicating the changes in transport signals are distinctly different from magnetization. Furthermore, the peak positions of the residual signals match the peak positions of $-d\rho_{yx}^{\mathrm{norm}}/dH$, indicating the source of residual signal is indeed transport and not magnetization. Also, the smoothly varying magnetization

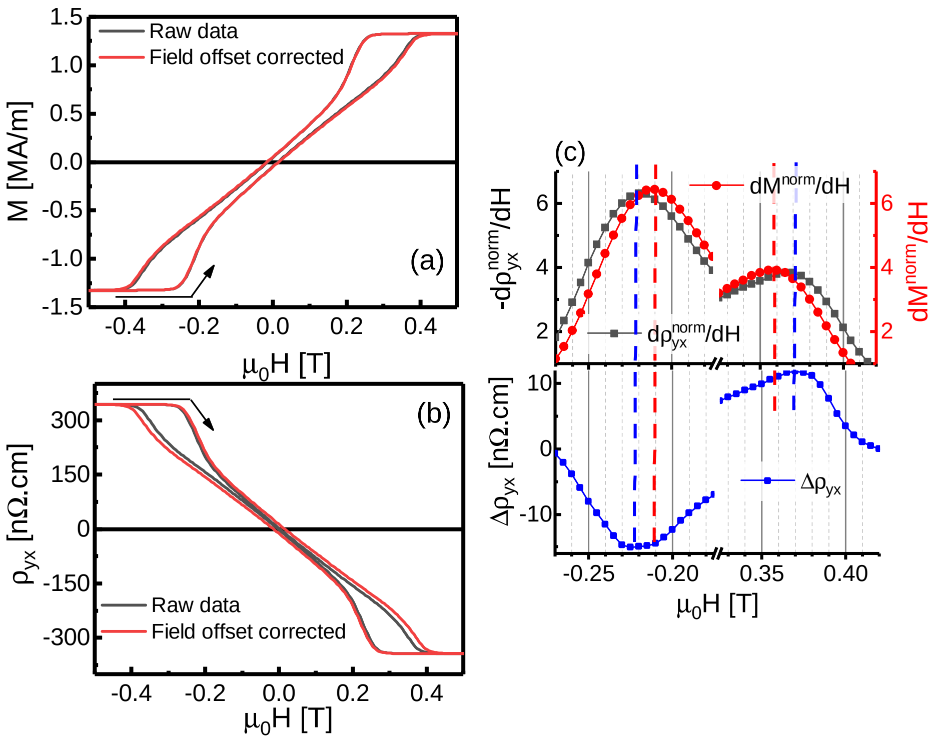

SUPP. FIG. 3. Topological Hall analysis and its validity. **(a),(b)** As-measured (black curve) and magnetic-field offset-corrected (red curve) $M(H)$ and $\rho_{yx}(H)$ for a $[\mathrm{Ir}(1)/\mathrm{Fe}(0.5)/\mathrm{Co}(0.5)/\mathrm{Pt}(1)]_{20}$ stack, recorded at 5 K. **(c)** Comparison of first derivatives of Hall (filled black squares) and magnetization (filled red circle) with the residual Hall signal. As indicated by blue dashed lines, the peak positions of the residual Hall signal (bottom panel, filled blue squares) match the peak positions of $-d\rho_{\mathrm{yx}}^{\mathrm{norm}}/dH$. While the red dashed lines indicate the peak position of dM^{norm}/dH, which is offset from the transport data. [The arrows in panels (a),(b) indicate the field sweep direction for $-H_s$ to $+H_s$ branch corresponding to panels (c). Panel (b) is reproduced in Fig. 1(a) in the main text.]

and Hall signal in this case provide reliable means of fitting.

B. Scaling of ρ_{yx} with ρ_{xx} and validity of the topological Hall effect analysis

Supplementary Fig. 4 shows examples of the extracted THE signal for multilayer stacks with varying number of repeats. Panels (a) and (d) show the measured ρ_{yx} and the fit [with and without accounting for the anomalous Hall effect (AHE) dependence on the specific shape of $\rho_{xx}(H)$]. Panels (c) and (f) show the overlap of THE signal resulting from the two

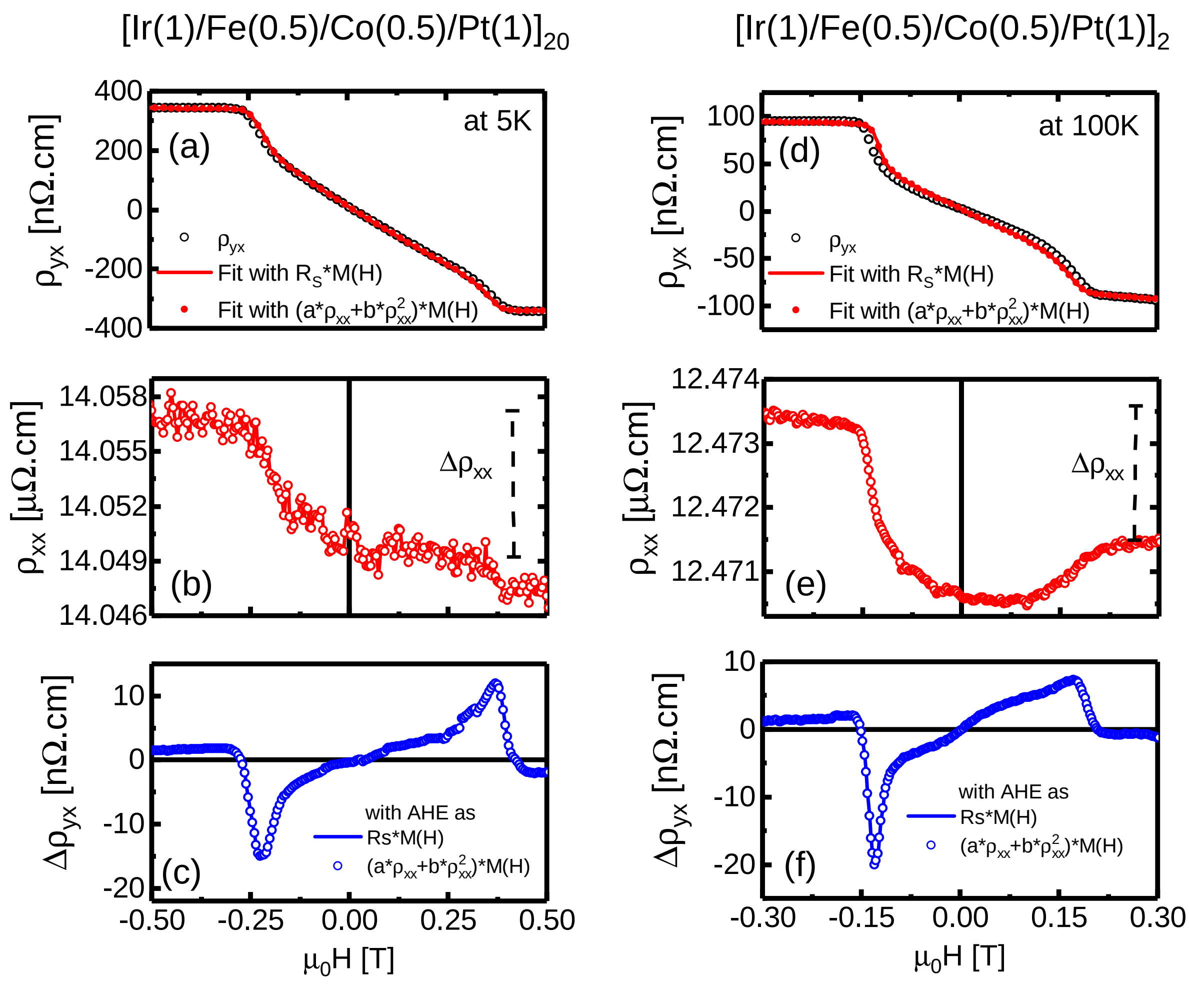

SUPP. FIG. 4. The role of longitudinal resistivity (ρ_{xx}) in the anomalous Hall effect (ρ_{yx}) and the extracted residual signal for $[\mathrm{Ir}(1)/(\mathrm{Fe}0.5)/\mathrm{Co}(0.5)/\mathrm{Pt}(1)]_{20}$ measured at 5 K and for $[\mathrm{Ir}(1)/(\mathrm{Fe}0.5)/\mathrm{Co}(0.5)/\mathrm{Pt}(1)]_{2}$ measured at 100 K. **(a,d)** ρ_{yx} as a function of $\mu_0 H$. Black open circles show the raw data, solid red line is a fit with AHE contribution taken as $R_s M(H)$, solid red circles are a fit with AHE as $a\rho_{xx} + b\rho_{xx}^2$. **(b,e)** ρ_{xx} as a function of $\mu_0 H$. **(c,f)** The residual between raw data of ρ_{yx} and fits. Solid line is the fit with AHE contribution taken as $R_s M(H)$, open circles are the fit with AHE as $a\rho_{xx} + b\rho_{xx}^2$.

sets of analysis. This result is consistent with the scaling behavior shown in Supp. Fig. 5. We note that the variation of $\rho_{xx}(H)$ is very small ($2 - 5$ n$\Omega \cdot$cm) in these multilayers for field values corresponding to magnetic textures. Furthermore, ρ_{xx} features asymmetry with field sweep [$\Delta\rho_{xx}$ shown in panels (b) and (e) points to the difference in ρ_{xx} for a positive and negative saturation of magnetization]. Such asymmetry in ρ_{xx} in multilayers can be

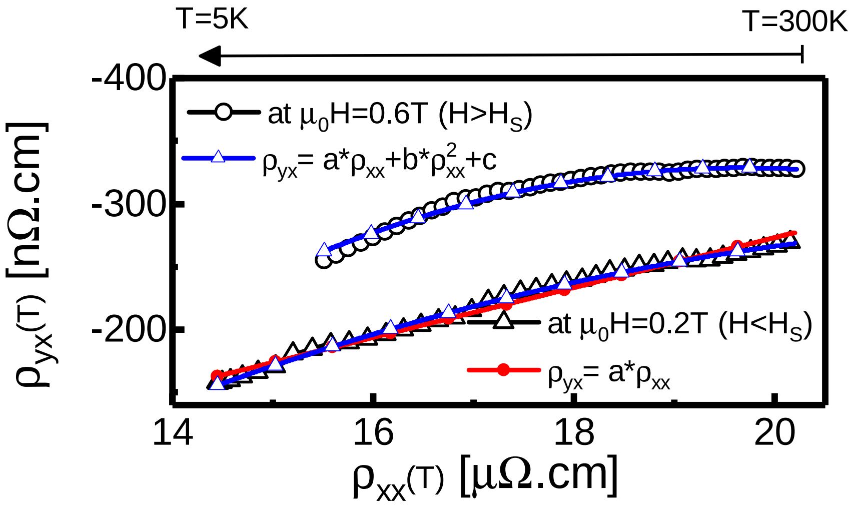

SUPP. FIG. 5. Scaling of $\rho_{yx}(T)$ with $\rho_{xx}(T)$ for $[\mathrm{Ir}(1)/(\mathrm{Fe}0.5)/\mathrm{Co}(0.5)/\mathrm{Pt}(1)]_{20}$ at fixed field .

attributed to the chiral domain walls stabilized by DMI [10]. However, the impact of such chiral domain wall resistance on ρ_{yx} is not clearly understood yet. Supplementary Fig. 4 clearly shows that $\rho_{xx}(H)$ has no effect on our extraction of THE.

The anomalous Hall effect in B20 systems, like FeGe, is well described by linear [$\rho_{xx}(H)$, skew] and quadratic [$\rho_{xx}^2(H)$, side jump and intrinsic] terms [11, 12], with transport usually dominated by the side jump mechanism for pure FeGe, and skew scattering when doped [11]. However, the scaling of ρ_{yx} with ρ_{xx} is not well understood in multilayers [13] due to the inhomogeneous structural and magnetic environment experienced by charge carriers. The scaling behavior in multilayers is expected to be controlled by the relative thickness of the layers and the mean free path of the charge carriers (l). When the layer thickness is less than l, transport is dominated by skew scattering [13]. This is consistent with the description of AHE by $R_S M(H)$, Eq. 3 of the main text, generally employed for magnetic multilayers [14, 15].

Supplementary Figs. 5 and 6 show the variation of ρ_{yx} and ρ_{xx} with temperature, field and the scaling behavior for different field values corresponding to skyrmions, chiral domains (at $H < H_S$) and a fully polarized ferromagnetic state ($H > H_S$). The figures show that within the field and temperature regimes explored in this study, the scaling between ρ_{yx} and ρ_{xx} can be approximated by a linear dependence. This is consistent with the dominant skew scattering expected in these multilayer systems [13]. A quadratic dependence is followed for

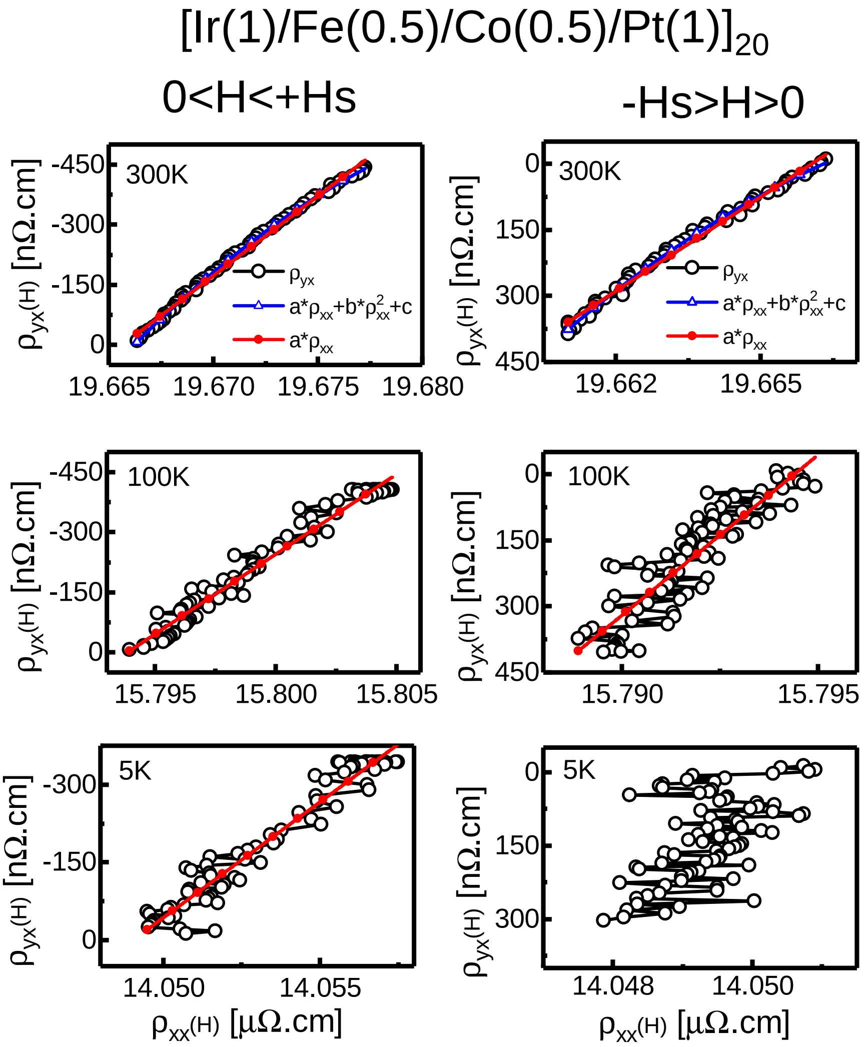

SUPP. FIG. 6. Scaling of $\rho_{yx}(H)$ with $\rho_{xx}(H)$ with applied field below the saturation of magnetization for [Ir(1)/(Fe0.5)/Co(0.5)/Pt(1)]$_{20}$ at different temperatures. Top row were measured at 300 K, middle row at 100 K and bottom row at 5 K.

a fully polarized ferromagnetic phase, consistent with a conventional ferromagnetic behavior. These observations indicate that skew scattering plays a major role in charge transport in the presence of chiral domain walls and skyrmions.

C. Temperature Dependence of the Topological Hall Effect

Supplementary Fig. 7 shows the temperature dependence of the magnetization, Hall, residual Hall profile and fit parameters. Supplementary Fig. 7(b) shows the experimental

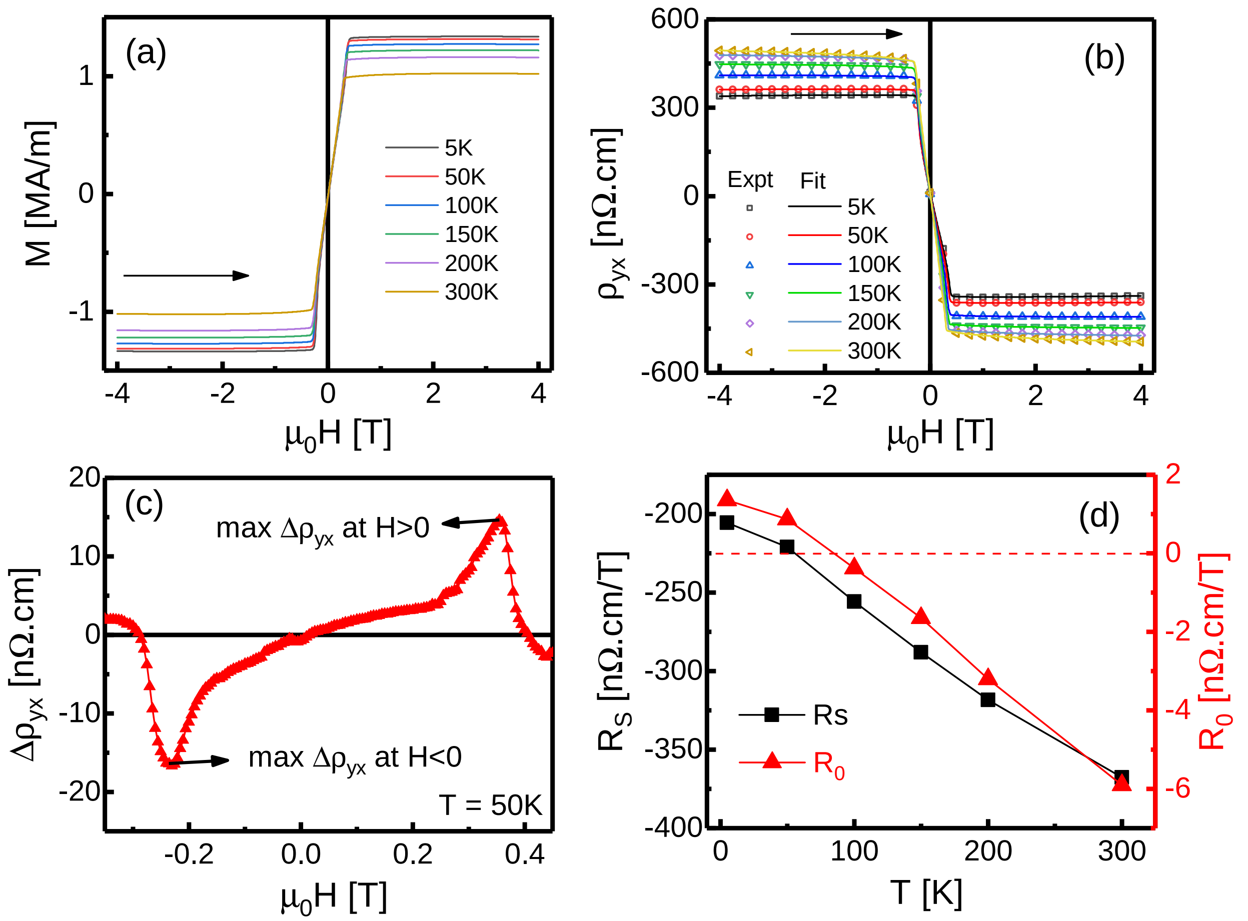

SUPP. FIG. 7. Temperature dependence of magnetization and Hall effect for [Ir(1)/Fe(0.5)/Co(0.5)/Pt(1)]$_{20}$. **(a)** Magnetization measured at various temperatures ($5-300$ K). **(b)** Total Hall signal measured at various temperatures ($5-300$ K) and the fit using the procedure described in the main text and data from panel (a). **(c)** Residual Hall signal at 50 K resulting from panel (b). **(d)** Temperature dependence of the anomalous Hall coefficient, R_S and the ordinary Hall coefficient, R_0, resulting from the fit data in panel (b) (R_0, is reproduced in Fig. 2(b) in the main text).

Hall data and the fit using Eq. 3 from the main text for different temperatures. Supplementary Fig. 7(c) shows the residual signal for $T = 50$ K resulting from panel (b), the arrows indicate the THE peak magnitude for $H < 0$ and $H > 0$. The magnitude of THE profiles for various temperatures resulting from panel (b) is summarized in Fig. 2(b) in the main text. The asymmetry in peak magnitude ($H < 0$ vs $H > 0$) results from hysteretic domain formation as the magnetization changes from negative saturation to positive upon field reversal [1].

As indicated in Supp. Fig. 7(b), the high field slope of the transport data representing the ordinary Hall coefficient, R_0, changes systematically as the temperature changes from 5 K to 300 K. Supplementary Fig. 7(d) summarizes the fit parameters $R_s(T)$ and $R_0(T)$, which is also reproduced in Fig. 2(b) in the main text together with $\max(|\Delta\rho_{yx}|)$. Importantly, $R_0(T)$ changes sign near $T = 85$ K, while $R_S(T)$ and $\max(|\Delta\rho_{yx}|)$ show that they scale differently with T. This suggests that the anomalous and topological components originate from different Berry phase mechanisms and that $\Delta\rho_{yx}$ is not a spurious signal associated with magnetization or the AHE component.

D. Evolution of the Topological Hall and Magnetic Textures at 50 K

Supplementary Fig. 8 shows the evolution of the residual Hall signal with field and the signed density of topological charge of various magnetic textures observed in the MFM scans at 50K. The analysis of skyrmions, $n_{\rm sk}$, worms, $n_{\rm w}$ and the total topological charge, $n_{\rm T}$, is similar to the 5K data presented in Fig. 3(a) in the main text. We observe qualitative agreement between $n_{\rm T}$ and $\Delta\rho_{yx}$ at 50 K, similar to the 5 K case. However, close inspection shows an offset between the $H > 0$ peak-positions for $\Delta\rho_{yx}$ and $n_{\rm T}$. This offset is, at least partially, a result of domain metastability and the perturbing influence of the magnetic tip, which can write and erase features. The metastability of these magnetic textures is demonstrated by performing repeated MFM scans on the same area of the sample. The data point corresponding to the red asterisk in Supp. Fig. 8 indicates the reduction in $n_{\rm T}$ as the tip erases the magnetic textures. We discuss this metastability in Supplementary Note 4 E.

E. Metastability and Manipulation of Magnetic Textures

In Supp. Fig. 9, we show examples of tip manipulation of domains highlighted in Supp. Fig. 16(d),(q) [also shown in main text Fig. 1(d),(j)]. Near the negative $\Delta\rho_{yx}$ peak, the perturbation from the tip creates more domains, reducing the net magnetization of the sample towards zero. On the other hand, near the positive $\Delta\rho_{yx}$ peak [Supp. Fig. 9(c),(d)], the perturbation deletes domains and pushes the system towards the polarized state. In these manipulations the influence of the tip cannot be simply ascribed to the stray field

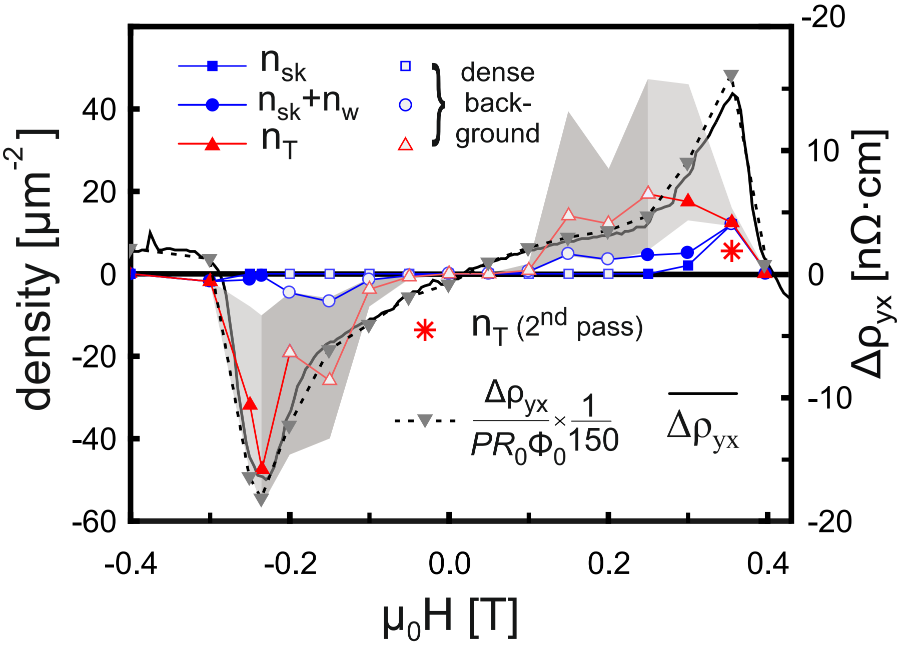

SUPP. FIG. 8. Evolution of magnetic textures and the residual Hall signal with field recorded at 50 K. Comparison between $\Delta\rho_{yx}$ (right axis) and the signed density of topological charge (left axis), which is determined from MFM data as that of skyrmions, $n_{\rm sk}$, or isolated features $n_{\rm sk}+n_{\rm w}$, or total topological charge, $n_{\rm T}$. For $n_{\rm T}$, each worm is assigned an appropriate number of skyrmions $|Q_{\rm W}|$ by fit, and each isolated skyrmion is counted once. We also show the density extracted from Eq. 2 in the main text assuming $\rho_{\rm TH} = \Delta\rho_{yx}$ (inverted gray triangles with dotted line), with $P = 0.56$ [16] and R_0 from Fig. 2(b). Empty symbols indicate points with a lower confidence, that result from counting worms embedded in a dense domain background using a deep learning network presented in the following sections. The gray shaded area shows the confidence bounds for $n_{\rm T}$, with uncertainty arising from the fit procedure of assigning skyrmions to worms detailed in the following sections. The confidence bounds from fitting worms embedded in the dense domain background appear with a darker shade. The red asterisk corresponds to a second pass over the scan area in which the tip erased magnetic features and reduced $n_{\rm T}$ [see e.g. Supp. Fig. 9(c),(d)].

because in both cases it is parallel to the external field directly under the tip, where it is strongest. This indicates that the perturbation from the tip drives the magnetic texture to a lower energy state, implying the metastability of these textures.

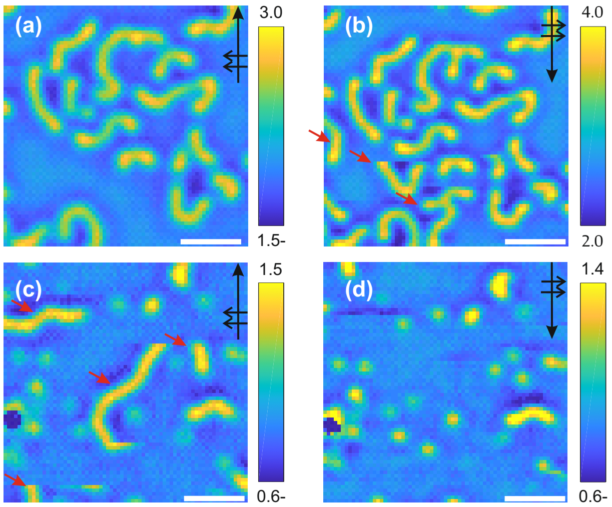

SUPP. FIG. 9. Examples of metastability and influence of the MFM tip on magnetic texture. Color bars show $\Delta f_{\mathrm{range}}$. Black arrows indicate the raster pattern of the scans: horizontal double arrows show the back or forth direction (fast axis); large vertical arrow shows the direction the tip is stepped in after a back and forth cycle (slow axis). **(a)** Detail from Supp. Fig. 16(d) [Fig. 1(d) in the main text] – part of a sweep towards $\mu_0 H = 0$ T – which shows the MFM signal acquired by a first pass at $h = 40$ nm. **(b)** The same area after a second MFM pass. Red arrows mark new features. **(c)** Detail from Supp. Fig. 16(q) [Fig. 1(j) in the main text] – part of a sweep from $\mu_0 H = 0$ T – which shows the MFM signal acquired by a first pass at $h = 45$ nm. Red arrows mark features that are missing in the next panel. **(d)** The same area as in (c) after a second MFM pass. Scalebars are 500 nm.

Supplementary Note 5. MICROMAGNETIC SIMULATIONS

Micromagnetic simulations of magnetic textures in [HM(1 nm)/FM(1 nm)/HM(1 nm)]$_{20}$ multilayers were performed using the MuMax^3 package [17], which accounts for interfacial DMI. HM and FM represent non-magnetic heavy metal (Pt and Ir) and ferromagnetic metal (Fe/Co) layers respectively. Stacks were modeled with a mesh size of $4 \times 4 \times 4$ nm^3 over an area of 2×2 μm^2. The magnetic parameters used for simulations [1, 18] are discussed in Supplementary Note 2. To simulate the evolution of magnetic textures, the stacks were relaxed to an equilibrium magnetic state at $\mu_0 H = 0$ T and then H was swept incrementally towards the saturation field. The simulations were performed at 0 K, while the magnetic parameters used correspond to $M(H)$ as determined by SQUID at 5 and 200 K. As presented

in Supp. Figs. 10, 11, the simulated magnetic textures evolve from stripe-domains at $\mu_0 H = 0$ T to worms and skyrmions at higher field. At sufficiently high field a uniformly magnetized FM state is realized. The evolution of the magnetic textures is qualitatively consistent with our experimental observations.

A. Simulated MFM Images

The MFM images presented in the main text and in Supp. Figs. 10, 11 were obtained by convolving the transfer function of the tip with the calculated stray field from the simulation magnetization [19]. The stray field was calculated using [20]:

$$H_z(\vec{k}) = -\frac{1}{2}\sum_{i=1}^{20}(1-e^{-kd_i})e^{-kh}\begin{pmatrix} -ik_x \\ -ik_y \\ 1 \end{pmatrix}\cdot \vec{m}_i(\vec{k}), \tag{1}$$

where $H_z(\vec{k})$ is the Fourier transform along the $x-y$ directions, with $\vec{k} = (k_x, k_y)$, $k \equiv \sqrt{k_x^2 + k_y^2}$, h is the distance between the MFM tip and the surface, d_i is the distance between the i-th layer and the surface and $\vec{m}_i(\vec{k})$ is the Fourier transform of the simulated magnetization in the i-th layer. To account for the blurring effects of the tip and amplitude we have used the model and tip parameters from [6]. Since the MFM does not measure in 4×4 nm^2 resolution, we have down-sampled the images so that each pixel covers a 24×24 nm^2 area, which reflects the sampling intervals of the experimental measurements.

B. Topological Charge of Simulated Worms

Figures 4(c),(f) in the main text present the contribution of individual domains to n_{T}. Equation 1 in the main text is an integral over the whole plane, which gives the total winding number for the whole magnetic texture. In order to obtain local information which reflects the contribution of an individual domain to this winding number we have to define domain boundaries. For a background polarized to $n_z = 1$ and domains at the center of which $n_z = -1$ we define the boundary by the line along which $n_z \approx 0.85$. We then performed the same integration as in Eq. 1, but only inside the domain boundaries. Our integration yielded results which were close to integer, indicating that the contribution to the integral from areas outside the domains was not significant. This analysis enabled us to provide an

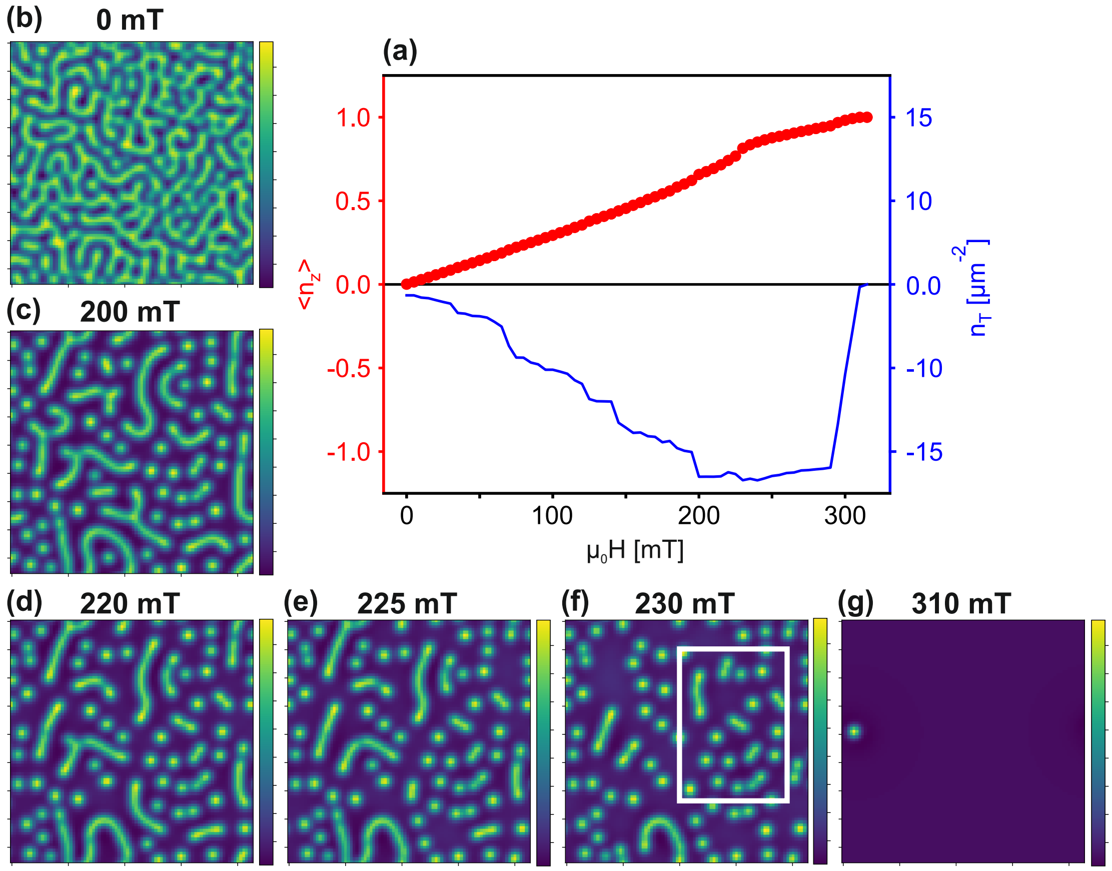

SUPP. FIG. 10. Results of micromagnetic simulations with $A = 11$ pJ/m, $D = 2$ mJ/m^2, $M_s =$ 1.34 MA/m and $K_{\text{eff}} = 0.2$ MJ/m^3. **(a)** Values of the mean magnetization along the z direction $\langle n_z \rangle$ (left axis, red circles), and of the topological charge density (right axis, blue line), calculated from the simulation results for various fields, considering only the bottom layer. **(b-g)** MFM images calculated from the simulation results showing the evolution of magnetic texture as the field progresses. The images are of a 2×2 μm^2 area. Color bars represent a range of approximately 0.2 Hz. White frame in (f) shows the features presented in Fig. 4 **(a-c)**

estimate for the contribution of individual domains to, n_{T}, the total topological charge in a simulated image.

Supplementary Note 6. MFM RESULTS

MFM images recorded at different temperature (T = 5, 50, 100, 150, 200 K) and field are presented at the end of this supplement, in the appendix, starting on page 28.

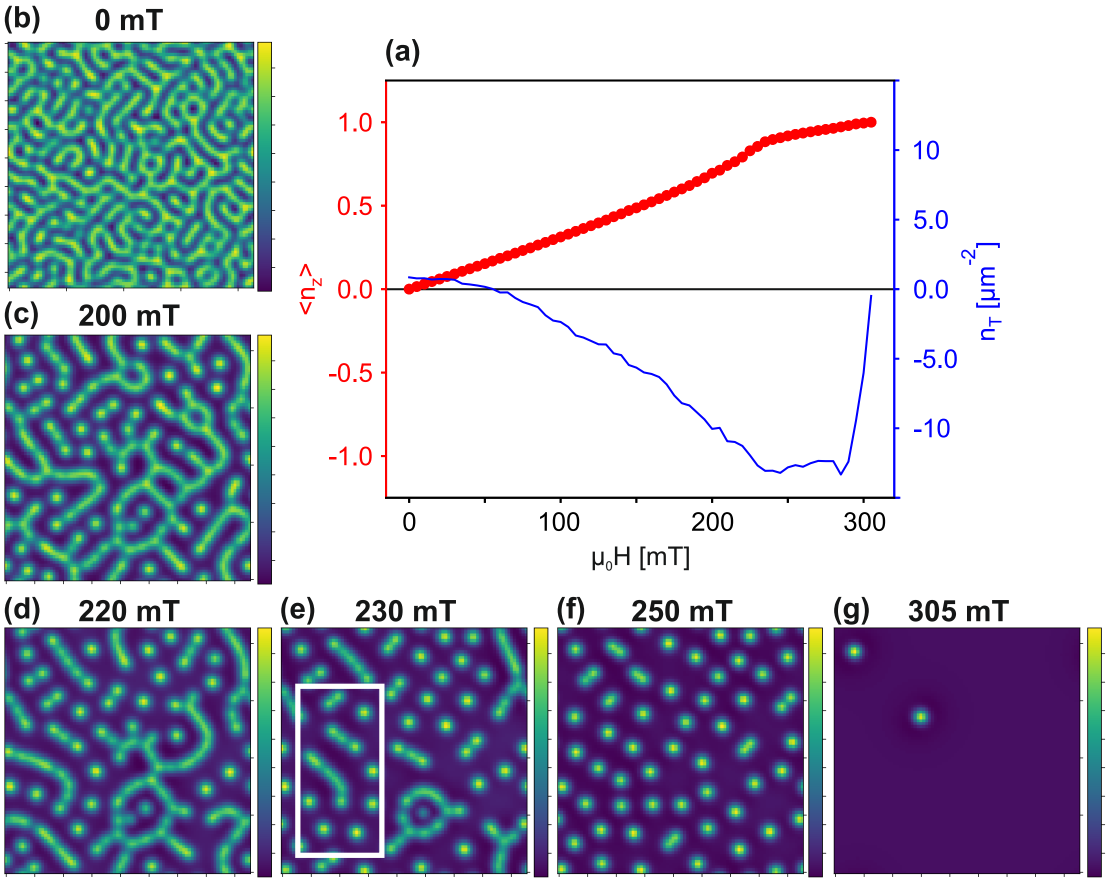

SUPP. FIG. 11. Results of micromagnetic simulations for $A = 11$ pJ/m, $D = 2$ mJ/m^2, $M_s = 1.16$ MA/m and $K_{\text{eff}} = 0.05$ MJ/m^3. **(a)** Values of the mean magnetization along the z direction $\langle n_z \rangle$ (left axis, red circles), and of the topological charge density (right axis, blue line), calculated from the simulation results for various fields, considering only the bottom layer. **(b-g)** MFM images calculated from the of the simulation results showing the evolution of magnetic texture as the field progresses. The images are of a $2 \times 2\mu$m^2 area. Color bars represent a range of approximately 0.2 Hz. White frame in (e) shows the features presented in Fig. 4 **(d-f)**

A. MFM Scans Near the Peaks of $\Delta\rho_{yx}$

The fields in which $\Delta\rho_{yx}$ peaks are of particular interest, as those are the fields where we expect to image magnetic textures which provide the maximal contribution to THE. Therefore, for each temperature we took care to obtain an MFM scan at those peaks. Supplementary Fig. 12 shows the magnetic texture measured at the negative peak and Supp. Fig. 13 for the positive one. As can be seen in Supp. Fig. 12, the lack of skyrmions and

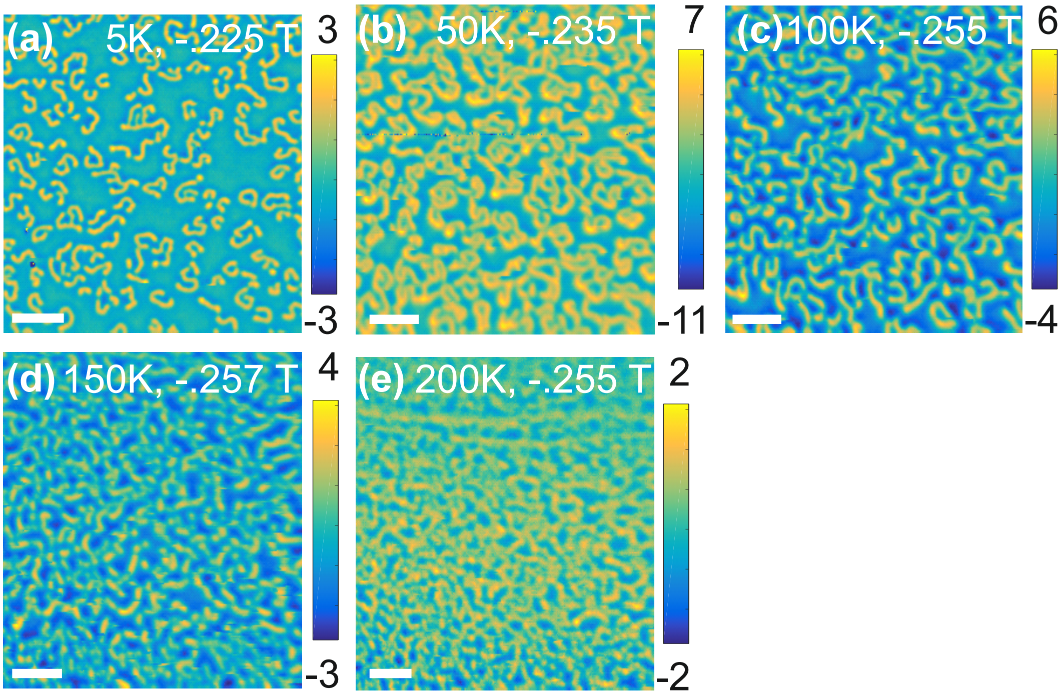

SUPP. FIG. 12. Temperature dependence of magnetic textures corresponding to the $\Delta\rho_{yx}$ peak for $H < 0$. **(a)-(e)** MFM images at T = 5, 50, 100, 150, 200 K and an applied field of $\mu_0 H = -0.225, -0.235, -0.255, -0.257, -0.255$ T respectively (scan heights are $40, 35, 35, 35, 30$ nm). Changes in field value represents the shift in peak position with temperature. Scalebars are 1 μm.

abundance of worms at the peak persists for $T \leq 100$ K. Above that temperature, magnetic imaging shows worms and skyrmions together, as in Supp. Fig. 13. Since the overall peak shapes of $\Delta\rho_{yx}$ are quite independent of temperature, we see this as further evidence for the higher topological charge of worms.

Supplementary Note 7. IMAGE ANALYSIS

As described in the main text, to estimate the topological charge of a worm (Q_{W}) we need to estimate the number of skyrmions it is composed of. Here, our analysis involves two steps. First, we label the magnetic worms based on strength of the MFM signal. Pixels exhibiting stronger positive MFM signal are more likely to be part of a worm, while the weak or negative ones correspond to the background. Here, we note that as the worms/skyrmions are magnetized in the opposite direction compared to the background and hence to the

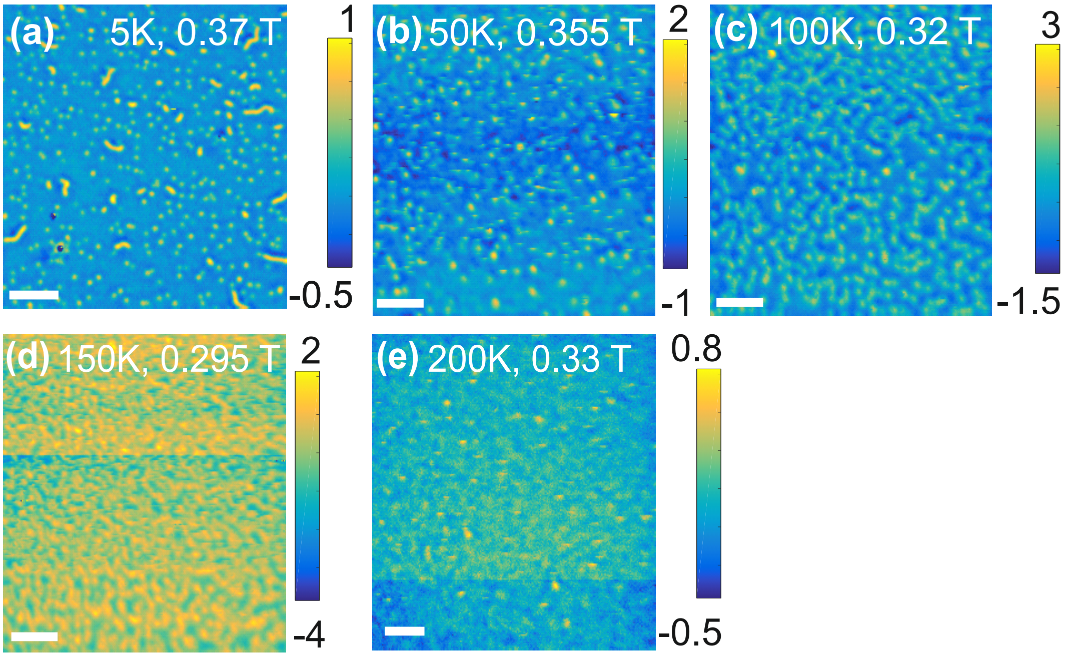

SUPP. FIG. 13. Temperature dependence of magnetic textures corresponding to the $\Delta\rho_{yx}$ peak for $H > 0$. **(a)-(e)** MFM images at T = 5, 50, 100, 150, 200 K and an applied field of $\mu_0 H$ = $0.37, 0.355, 0.32, 0.295, 0.33$ T respectively (scan heights are $40, 35, 35, 50, 40$ nm.). Change in field value represents the shift in peak position with temperature. Scalebars are 1 μm.

MFM tip, the tip experiences a repulsive force giving rise to a strong positive signal. Such a strong signal from worms/skyrmions makes the identification easier. After labeling, the second step involves the fitting of each worm with a train of skyrmions. For each connected group of pixels labeled as "worms", we perform several iterations and attempt to find the optimal number of skyrmions which best describes the data.

A. Labeling

We start by applying the K-means clustering algorithm [21] on the Δf values of a scan in order to divide the data into two groups – worms (including skyrmions) and background. We denote the mean value for worms (the stronger signal) as c_1 and the background as c_2 (weaker signal). Each pixel in the image is thus attributed a value c_α with $\alpha = 1$ or $\alpha = 2$, according to the group it belongs to.

Due to the effects of tip convolution and scan height, the magnetic features measured by the MFM are larger than their true size. Therefore, the smallest domain sizes measured by MFM are limited by the tip curvature radius of ≈ 30 nm, which is approximately the size of a pixel in our images 25×25 nm^2 . Also, based on our previous work [1, 6], skyrmions with a radius of 40 nm are a reasonable lower limit. We feed this spatial information into our labeling analysis to avoid features smaller than two pixels, which are not physical.

The optimal labeling, given these two conditions, minimizes the following cost function: $J(f) = \sum_i' D_i^1(f) + \sum_i'' D_i^2(f) + \sum_{\langle i,j \rangle} V_{ij}$, to find the optimal matrix of labels f. Here i, j are pixels in the image, $\sum_i'$ is a sum over all pixels labeled c_1, $\sum_i''$ is a sum over all pixels labeled c_2, $D_i^\alpha = (\Delta f_i - c_\alpha)^2$ is a measure for how well the label α fits the ith pixel, $V_{ij} = \lambda$ if $f_i \neq f_j$ and 0 otherwise, and $\sum_{\langle i,j \rangle}$ is a sum over nearest neighbors. The minimization of $J(f)$ is performed using graph cuts with a piecewise constant prior [22]. The value of the parameter λ is chosen as the smallest parameter for which the optimal labeling no longer includes single-pixel domains. The resulting labeling f is a binary image which is true for pixels positively identified as worms, and false for those in the background.

B. Fitting

For each connected group of pixels labeled as worms, we now fit the number of skyrmions. We perform several iterations, assuming a different minimal spacing between skyrmions in each one. Similarly, K-means was employed to iteratively divide the worm pixels into groups, where the mean of each group is assumed to be the location of a skyrmion. If the mean of the group lies outside the worm area (as would happen with a small number of groups or banana shaped worms), we choose the closest point within the worm to be the new mean for the group. The worm is divided into an increasing number of groups until at some number N, the spacing between groups is smaller than the minimal spacing. We then fit $N-1$ skyrmions, with the group means used as initial skyrmion positions. The fitting is done using a multipole expansion for the magnetic field from skyrmions (MEFS) model [6], in which we approximate the stray field from a skyrmion as the field of a magnetic dipole and quadrupole. Since we are only interested in the number of skyrmions which fits the data, we employ the truncated cone model [6] for the tip because it is computationally cheap. Skyrmion–skyrmion repulsion grows exponentially on the scale of the skyrmion radius [23],

therefore we reject fit results in which the distance between neighboring skyrmions is smaller than a skyrmion radius, which we previously estimated to be approximently 40 nm [6].

In order to provide some constraint on the amplitude, we first consider the case of a single skyrmion; as we have stated before, a single skyrmion will be captured by several pixels. Given the sharp shape of the skyrmion signal, the peak will be larger than the mean value of all the pixels which constitute the skyrmion. Extending this condition to worms, we constrain the amplitude of each fitted skyrmion to be at least the mean value of all pixels labeled as worms.

The fitting procedure that we establish therefore assigns the minimum number of skyrmions which best fits the data by constraining the amplitude per skyrmion. As a lower bound we take the numbers of skyrmions which can fit in a worm, and are similar in width to skyrmions observed in Fig. 1(j) ($\approx$ 100 nm) since if their densities were lower we should able to resolve them. For an upper bound we take the number of skyrmions which can fit into a worms so that their centers are still 40 nm apart which is the typical radius [6]. The lower bound thus corresponds to the width of skyrmions which we have measured, and the upper bound corresponds to our estimate of the actual skyrmion radius without tip convolution [6].

C. Worms in a Background of Dense Domains

As Supp. Figs. 16, 17, and 18 show, for fields in the intermediate range between $\mu_0 H =$ 0 T and THE peaks, the magnetic texture is dominated by dense domains, giving rise to an apparent non-zero Hall signal, $\Delta\rho_{yx} \neq 0$, as shown in Supp. Fig. 7(c). We would like to obtain some insight on whether or not this contribution to $\Delta\rho_{yx}$ comes from worm-like features, as was clearly the case for images corresponding to fields between the peaks in $\Delta\rho_{yx}$ and saturation. Our hypothesis is that as the dense stripe domain pattern evolves upon reducing the field towards $\mu_0 H = 0$ T, some of the worm-like features observed at higher fields persist and coexist with stripe domains and contribute to THE. In order to extract the density of worms and hence topological charge from these images, we need a vigorous method for classifying pixels to worms and dense domain background.

Here, classification which relies on the Δf value is problematic, as both up-domains and worms exhibit a similar positive signal. Establishing such a classification based on a

quantitative metric which will be reliable for a range of scans obtained over a wide variety of temperature, field and scans height using a set of predetermined features can be remarkably difficult, as such general features which distinguish a worm from a dense domain pattern are very hard to define. Here, we pursue a more data-oriented approach in which an algorithm extracts relevant features from supplied examples. We assume that a feature contributes to THE signal if it resembles a worm [such as in Supp. Fig. 16(d)] more than it resembles dense domains [such as in Supp. Fig. 16(g)] and hence the parameters corresponding to worms in Supp. Fig. 12(a) act as reference for classification of features in dense images.

Deep learning models, specifically convolutional neural networks, are especially well suited for this task [24], as they can learn to distinguish complex features accurately using a large number of successive convolution layers. In addition to classification, we require the model to output spatial information - specifically which pixels in the image correspond to worms and otherwise dense domains. As shown by Long et al. [25], it is possible to add several upsampling layers to a classification convolutional network in a directed acyclic graph (DAG) architecture, and thus obtain spatial information in addition to classification. In the data analysis for this paper, we have used the MatConvNet toolbox [26], which provides an implementation for DAG neural networks that can be used in Matlab. The classification network we used was the pretrained VGG-VD16 network, downloaded from the MatConvNet website. We adapted the algorithm for our purpose by changing the last fully connected layer to have 2 classes only, and adding upsampling layers with 2 classes for the FCN8 architecture [25].

In order to minimize the training time, we try to make maximal use of existing knowledge, and only fine-tune the network to our needs. The VGG-VD16 network was trained on RGB images. Since the measurement noise in our MFM is at least 10 mHz, and the maximum values we measure are smaller than 100 Hz, we require only four significant digits to accurately describe our data. We can therefore convert our MFM data (single channel in double-precision floating-point format) to RGB images (three channels of unsigned 8-bit) without any loss of accuracy. We achieve this by creating a histogram of the data with the number of bins adjusted so that the bin centers are 10 mHz apart (noise limit). Each bin was then assigned with a corresponding RGB value using the Matlab Parula color map, thus rendering an RGB image which contains all the information from the MFM scan. This routine is very similar to the way MFM images are displayed as RGB images in all figures

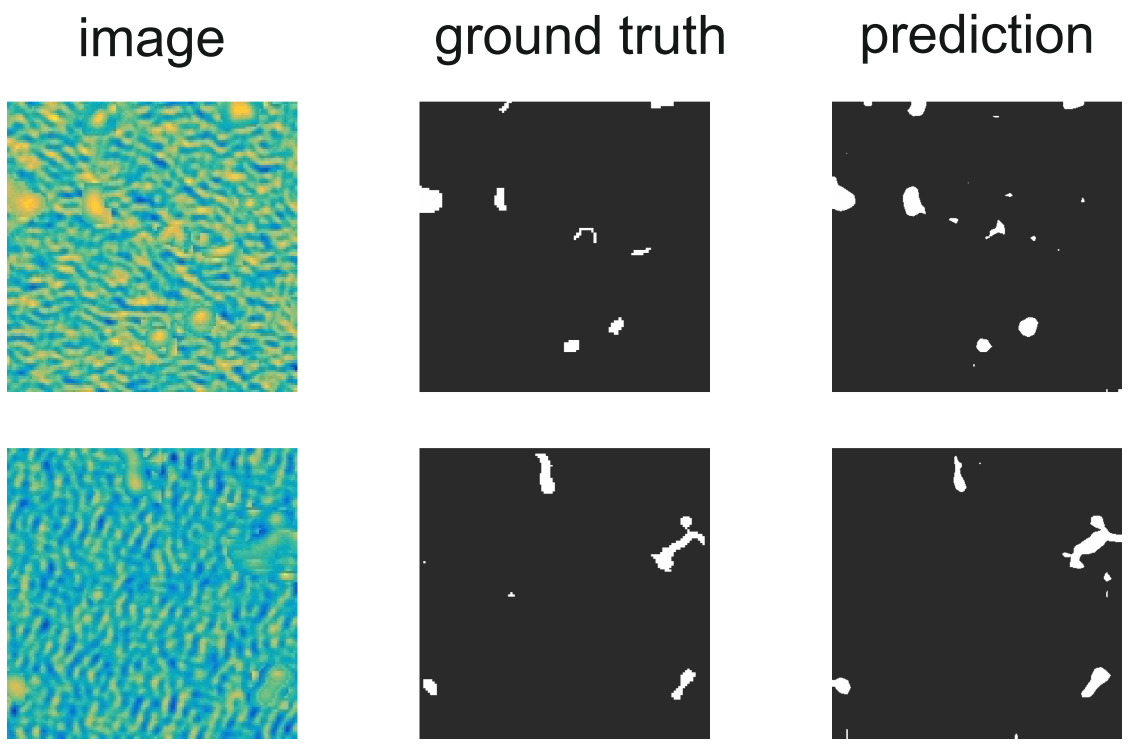

SUPP. FIG. 14. Examples for the application of the deep learning model on the test set. Left column shows the synthetic data of worms on a dense domain background. The middle column shows the ground truth used for training. Rightmost column is the prediction of the model. These images do not have a single length scale for all features, as different features come from different temperatures where the conversion form pixels to length is different.

in this paper.

Next, we need to create synthetic data which is representative of worms and dense domain pattern to properly train the model to segment any given image. For instance, learning the features of worms will require many different images to account for the great variations in signal intensity, length-scale and shape. On the other hand, it is also important to provide examples of dense domain patterns with different rotations to avoid over-fitting to orientations. Notably, the scaling portion of the training is well taken care of by MatConvNet which randomly rescales training images.

For training images of the dense domain background we took a set of images measured at $\mu_0 H = 0$ T, where the magnetization is roughly 0, hence we expect an equal number of up and down domains with no worms, and randomly cropped a 128×128 frame. In addition, the cropped image was randomly rotated by $0, 90, 180$ or 270 degrees. For worms

we used the algorithm elaborated in Supplementary Note 7 A on images with worms on a polarized background to obtain labeled examples. We then generated a list of all the worms in the images, where we define an individual worm as a set of pixels labeled as worms with an 8-connected neighborhood (pixels which are no more than one row and/or one column away from each other). For each dense domain background we then randomly picked about 10 worms from all the available images, and then planted each one in the dense domain background with a random margin of several pixels around it, so that the model will be able to capture the entire shape of the worms, and not just the stronger signal part.

The signal intensity of worms varies greatly between different scans, and can be much stronger than the signal of the dense domains. To avoid a bias towards those examples, we ensured all worms have the same maximum value. For scans of worms and dense domains that were obtained under similar conditions – temperature and scan height, the worms have a signal which is about $\times 4$ stronger than that of the dense domains [Supp. Fig. 17(c) and (h) for example]. We therefore constrained the absolute value of the mean of the dense domains in the synthetic image to be 0.3 of the worm maximum so that it will be similar to the raw data ratio.

Overall we have used 20000 images, 18500 as a training set and 1500 for validation. For each image we created both a classification and a segmentation training example. During training we used the MatConvNet [26] default parameters except for the class weights. As there are approximately 10 background pixels for each worm pixel, a model can reach high accuracy if it classifies all pixels as background. In order to avoid the convergence of the model to such a solution, we assigned a weight of 10 to worms and 1 to the background. After 4 epochs the model achieved an accuracy of 97% on 500 extra images used as a test set. Further training reduced the test set accuracy, probably as a result of over-fitting. The true negative rate was 0.98 and the true positive rate was 0.76. Two examples from the test set can be seen in Supp. Fig. 14.

Each image in the intermediate field range was then fed through the network. The output is a binary image which is true for worms and false for the background. An example for the network application on data can be seen in Supp. Fig. 15. We then copied the pixels classified as worms (with a margin of two pixels) from the original image and placed them in a background of zeros. A Gaussian filter of 5×5 pixels with $\sigma = 10$ was then used to smoothen the edges. The result is an estimate of a scan which has only the worms in it

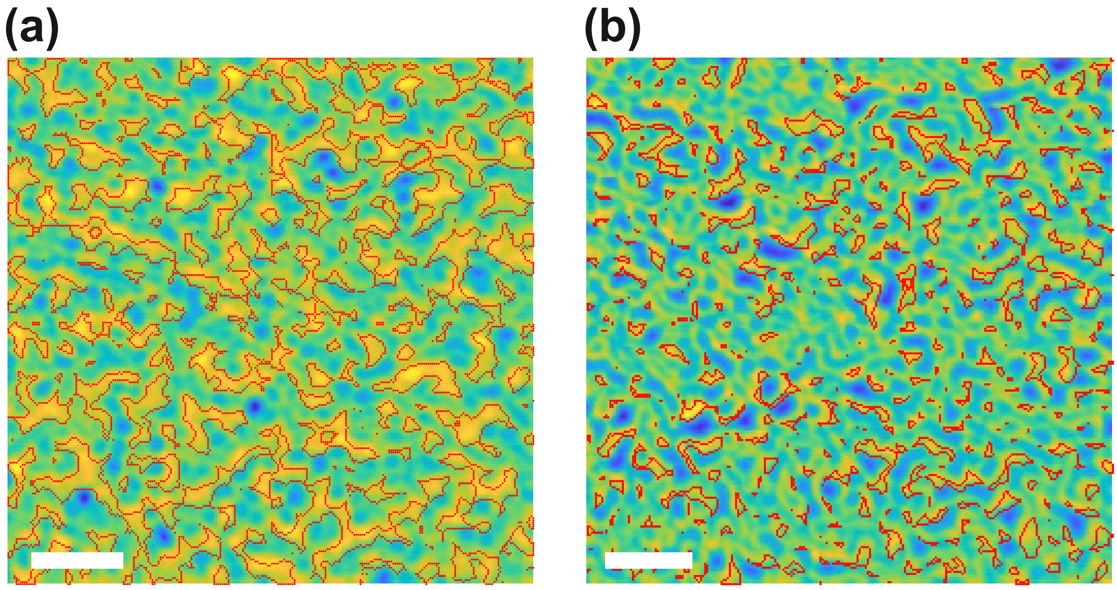

SUPP. FIG. 15. Examples for the application of the deep learning model on data. **(a)** network output on Supp. Fig. 16(g), **(b)** network output on Supp. Fig. 17(d). The red markers are the boundaries of areas classified by the network as worms. The scale bars are of 1 μm.

without the dense domain background. We then applied the algorithm in Supplementary Note 7 A and Supplementary Note 7 B on this image to obtain the topological charge.

Given the true negative and positive rates of 0.98 and 0.76 respectively, we proceeded to estimate the classification errors. Our estimate is based on the probability that the network will make a classification error for a single pixel. For simplicity, we will assume that the prediction of a pixel class is independent of other pixels, and that it follows a binomial distribution. For convolutional networks that assumption is not necessarily accurate, but it is sufficient for determining error bounds.

We can calculate an explicit upper bound for how many worms the network missed – let us assume a worst case scenario where the network has identified zero worms, therefore there are $N = 256^2$ pixels labeled as dense domains. The probability that the network will miss a worm is $p = 0.02$. The probability that the network missed more than 3000 pixels who should have been labeled as worms is thus negligible. From Supp. Fig. 17(a), $\approx$ 3000 pixels were labeled as worms, and this was the lowest density we measured. We therefore conclude that the density which can be attributed to worms the network missed is negligible for our data.

Applying the above calculation to pixels marked as worms, we note that there is a 0.95 probability that the fraction of pixels falsely marked as worms is ≈ 0.25. The lower bound for scans where the network was used to identify worms should thus be 0.75 of its fitted value.

Supplementary Note 8. ESTIMATE OF THE ORDINARY HALL COEFFICIENT

To get a rough estimate for the ordinary Hall coefficient in the absence of compensation resulting from electron and holes, we can assume a single charge carrier per unit cell [27]. Assuming a bulk value for the density ($n \approx n_{\mathrm{Fe}} \approx n_{\mathrm{Co}} \approx 4 \cdot 10^{22}$ cm^{-3}) we obtain: $R_0' \approx (ne)^{-1} \approx 15$ n$\Omega \cdot$cm/T. This is about $10 \times R_0(5\mathrm{K})$.

---

[1] A. Soumyanarayanan, M. Raju, A. L. Gonzalez Oyarce, A. K. C. Tan, M.-Y. Im, A. P. Petrović, P. Ho, K. H. Khoo, M. Tran, C. K. Gan, F. Ernult, and C. Panagopoulos, Nat. Mater. **16**, 898 (2017).

[2] T. R. Albrecht, P. Grütter, D. Horne, and D. Rugar, J. Appl. Phys. **69**, 668 (1991).

[3] In principle the force includes contributions from electrostatic forces and the van der Waals force, in addition to the magnetic force that we are after. We minimize the contributions of the undesirable forces by working far enough for the latter force to not contribute and by adjusting the tip-sample voltage to compensate for the tip-sample work function difference.

[4] We used two commercial probes by Team Nanotec, model ML3 ($35-40$ nm Co alloy coating), with $f_0 \approx 75$ kHz and $k_0 \approx 1$ N/m.

[5] F. J. Giessibl, Phys. Rev. B **56**, 16010 (1997).

[6] A. Yagil, A. Almoalem, A. Soumyanarayanan, A. K. C. Tan, M. Raju, C. Panagopoulos, and O. M. Auslaender, Appl. Phys. Lett. **112**, 192403 (2018).

[7] M. T. Johnson, P. J. H. Bloemen, F. J. A. d. Broeder, and J. J. d. Vries, Rep. Prog. Phys. **59**, 1409 (1996).

[8] R. Ritz, M. Halder, C. Franz, A. Bauer, M. Wagner, R. Bamler, A. Rosch, and C. Pfleiderer, Phys. Rev. B **87**, 134424 (2013).

[9] N. Kanazawa, M. Kubota, A. Tsukazaki, Y. Kozuka, K. S. Takahashi, M. Kawasaki, M. Ichikawa, F. Kagawa, and Y. Tokura, Phys. Rev. B **91**, 041122 (2015).

[10] Y. Yin, D.-S. Han, J.-S. Kim, R. Lavrijsen, K.-J. Lee, S.-W. Lee, K.-W. Kim, H.-W. Lee, H. J. Swagten, and B. Koopmans, Appl. Phys. Lett. **110**, 122401 (2017).

[11] C. S. Spencer, J. Gayles, N. A. Porter, S. Sugimoto, Z. Aslam, C. J. Kinane, T. R. Charlton, F. Freimuth, S. Chadov, S. Langridge, *et al.*, Phys. Rev. B **97**, 214406 (2018).

[12] N. Nagaosa, J. Sinova, S. Onoda, A. H. MacDonald, and N. P. Ong, Rev. Mod. Phys. **82**, 1539 (2010).

[13] S. Zhang, Phys. Rev. B **51**, 3632 (1995).

[14] K. Zeissler, S. Finizio, K. Shahbazi, J. Massey, F. Al Ma'Mari, D. M. Bracher, A. Kleibert, M. C. Rosamond, E. H. Linfield, T. A. Moore, J. Raabe, G. Burnell, and C. H. Marrows, Nat. Nanotechnol. (online) (2018), 10.1038/s41565-018-0268-y.

[15] D. Maccariello, W. Legrand, N. Reyren, K. Garcia, K. Bouzehouane, S. Collin, V. Cros, and A. Fert, Nat. Nanotechnol. **13**, 233 (2018).

[16] A. Rajanikanth, S. Kasai, N. Ohshima, and K. Hono, Appl. Phys. Lett. **97**, 022505 (2010).

[17] A. Vansteenkiste, J. Leliaert, M. Dvornik, M. Helsen, F. Garcia-Sanchez, and B. Van Waeyenberge, AIP Advances **4**, 107133 (2014).

[18] B. Satywali, F. Ma, S. He, M. Raju, V. P. Kravchuk, M. Garst, A. Soumyanarayanan, and C. Panagopoulos, arXiv:1802.03979 (2018).

[19] P. J. A. van Schendel, H. J. Hug, B. Stiefel, S. Martin, and H.-J. Güntherodt, J. Appl. Phys. **88**, 435 (2000).

[20] C. Schönenberger and S. F. Alvarado, Z. Phy. B **80**, 373 (1990).

[21] J. A. Hartigan and M. A. Wong, J. R. Stat. Soc. Series C **28**, 100 (1979).

[22] O. Veksler, *Efficient graph-based energy minimization methods in computer vision*, Ph.D. thesis, Cornell University (1999).

[23] Shi-Zeng Lin, C. Reichhardt, C. D. Batista, and A. Saxena, Phys. Rev. B **87**, 214419 (2013).

[24] Y. LeCun, Y. Bengio, and G. Hinton, Nature **521**, 436 (2015).

[25] J. Long, E. Shelhamer, and T. Darrell, in *Proceedings of the IEEE Conference on Computer Vision and Pattern Recognition* (2015) pp. 3431–3440.

[26] A. Vedaldi and K. Lenc, in *Proceedings of the 23rd ACM International Conference on Multimedia*, MM '15 (ACM, 2015) pp. 689–692.

[27] E. M. Pugh and N. Rostoker, Rev. Mod. Phys. **25**, 151 (1953).

APPENDIX –
MFM IMAGES AS A FUNCTION OF FIELD AND TEMPERATURE

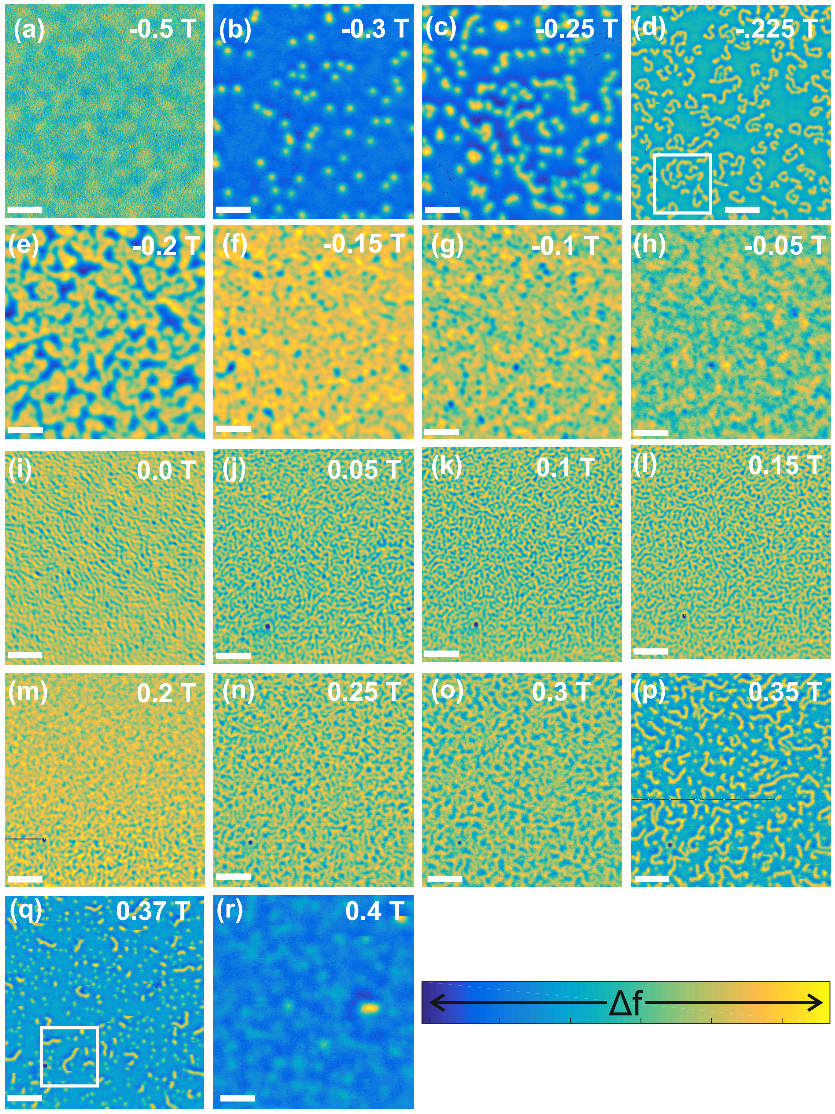

SUPP. FIG. 16. MFM scans at $T = 5$ K. The applied field is indicated on each panel. The scale bar is $1\,\mu$m. Scan heights for panels (a-r) are: $60, 76, 60, 40, 60, 65, 63, 60, 50, 58, 50, 90, 50, 50, 60, 50, 45, 60$ nm respectively. The color-bar indicates the range of Δf values. The for each scan the range was: $0.3, 0.7, 1.8, 6, 2.3, 1, 0.9, 0.7, 1.2, 1, 1.6, 2.4, 1.8, 2.5, 2.5, 1.2, 1.5, 1$ Hz. White frames in panels (d,q) indicate the areas from which panels (a,b) and (c,d) in Supp. Fig. 9 where taken from.

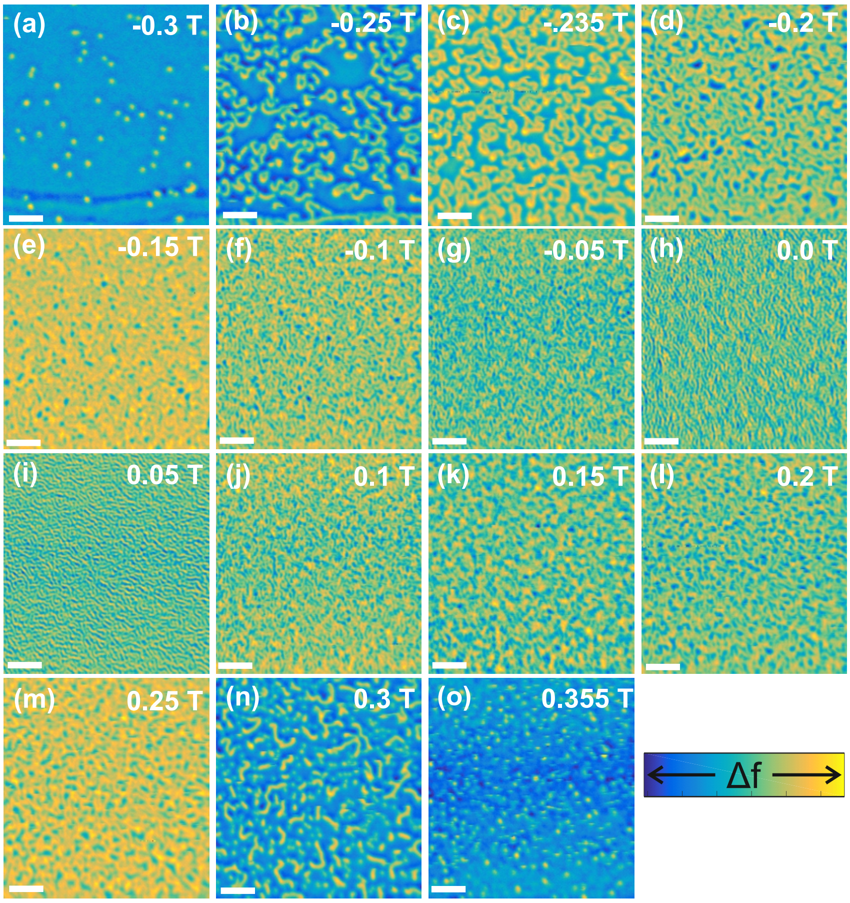

SUPP. FIG. 17. MFM scans at $T = 50$ K. The applied field is indicated on each panel. The scale bar is 1 μm. Scan heights for panels (a-o) are: $40, 50, 35, 40, 45, 50, 45, 35, 38, 35, 32, 32, 33, 36, 35$ nm respectively. The color-bar indicates the range of Δf values. For each scan the range was: $6, 11, 10, 11, 8, 5, 2.5, 2.25, 2.5, 5, 3, 10, 3$ Hz.

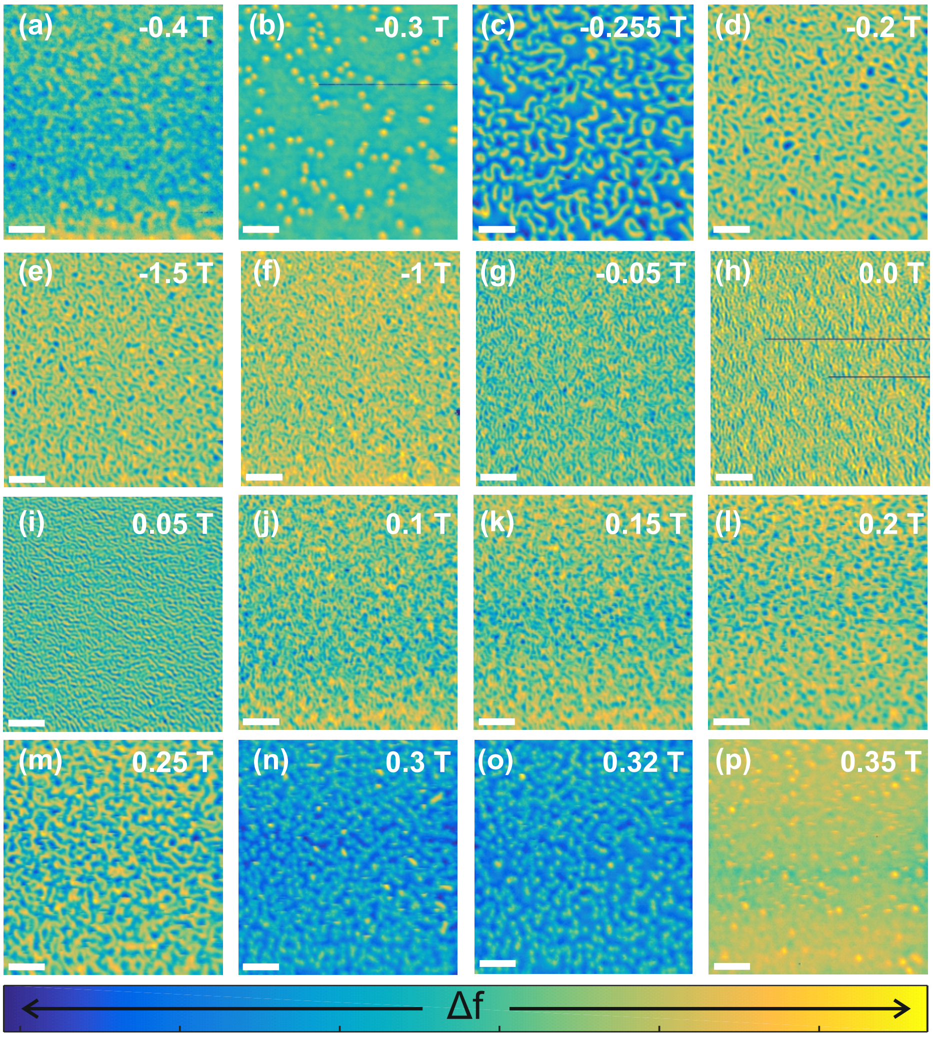

SUPP. FIG. 18. MFM scans at $T = 100$ K. The applied field is indicated on each panel. The scale bar is $1\,\mu$m. Scan heights for panels (a-p) are: $37, 32, 36, 36, 34, 34, 33, 33, 33, 32, 32, 33, 45, 36, 35, 35$ nm respectively. The color-bar indicates the range of Δf values. For each scan the range was: $1, 7, 10, 9, 9, 6, 4.2, 2.4, 2.25, 5, 7.2, 11, 9, 7, 4.5, 4.5$ Hz

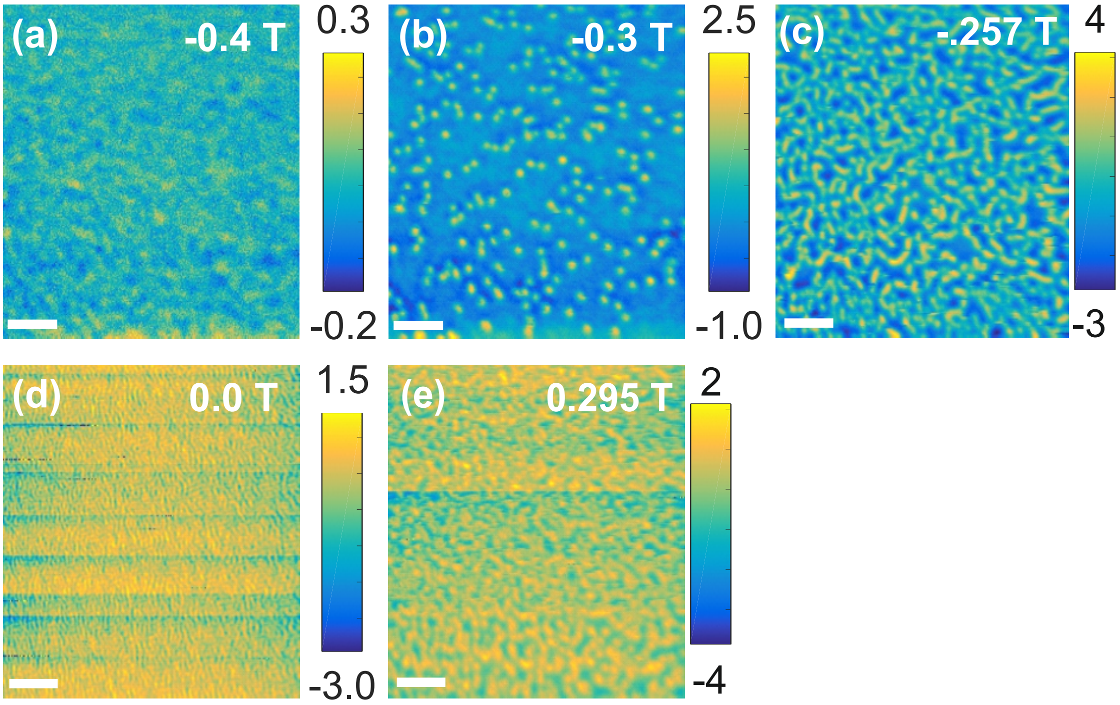

SUPP. FIG. 19. MFM scans at $T = 150$ K. The applied field is indicated on each panel. The scale bar is $1\,\mu$m. Scan heights for panels (a-e) are: $50, 50, 35, 36, 50$ nm respectively.

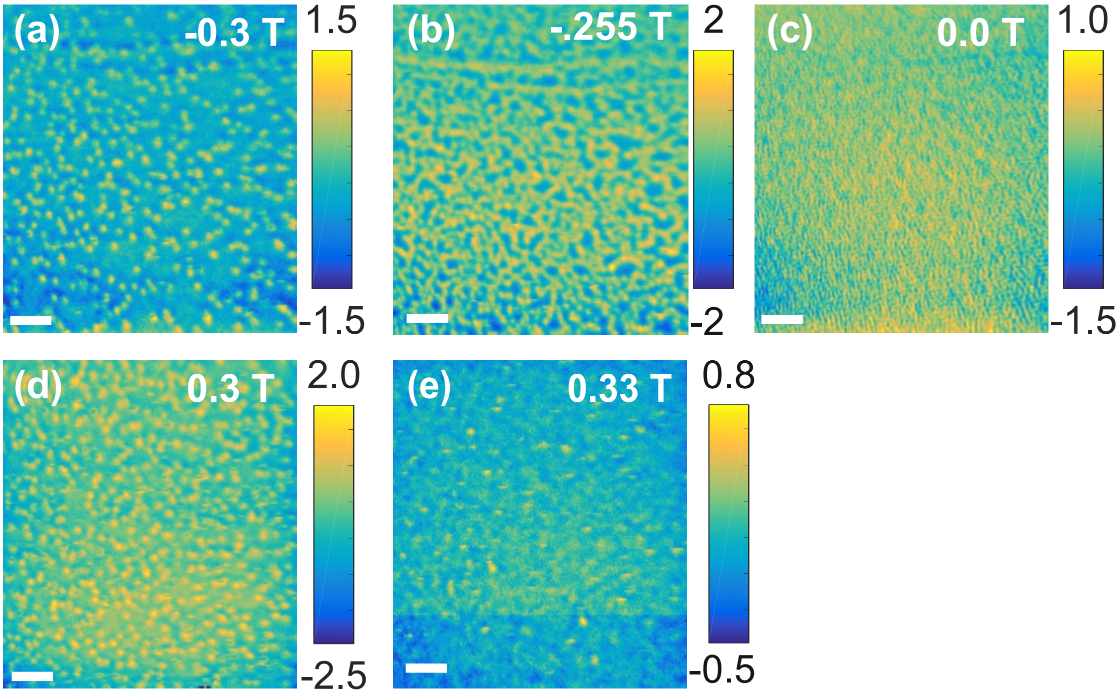

SUPP. FIG. 20. MFM scans at $T = 200$ K. The applied field is indicated on each panel. The scale bar is $1\,\mu$m. Scan heights for panels (a-e) are: $40, 30, 36, 40, 40$ nm respectively.
